# The influence of vaginal microbiota on ewe fertility: a metagenomic and functional genomic approach

**DOI:** 10.1186/s40168-025-02165-z

**Published:** 2025-08-01

**Authors:** Edgar L. Reinoso-Peláez, María Saura, Carmen González, Manuel Ramón, Jorge H. Calvo, Magdalena Serrano

**Affiliations:** 1 Instituto Nacional de Investigación y Tecnología Agraria y Alimentaria (INIA-CSIC), Ctra. de La Coruña, Km 7.5, 28040 Madrid, Spain; 2 Universidad Politécnica de Madrid, Escuela Técnica Superior de Ingeniería Agronómica, Alimentaria y de Biosistemas, Madrid, Spain; 3 Instituto de Investigaciones Marinas, Rúa Eduardo Cabello 6, Vigo, 36208 Spain; 4 ARAID-Centro de Investigación y Tecnología Agroalimentaria de Aragón (CITA)-IA2, Av. de Montañana, 930, Zaragoza, 50059 Spain

**Keywords:** Artificial insemination, Fertility, Metagenomics, Microbiome, Ovine, Reproductive success, Vaginal microbiota, Random forest, Microbiome-wide prediction

## Abstract

**Background:**

Despite advancements in artificial insemination, sheep fertility rates remain suboptimal. Recent studies in other species highlight the critical role of reproductive microbiota in influencing fertility outcomes. This research explores the relationship between ovine vaginal microbiota, associated functional pathways, and fertility using advanced nanopore long-reading metagenomic sequencing on 297 ewes from three Spanish breeds across four herds. The study aimed to describe a core vaginal microbiota, analyse the complex interactions with herd, breed, age, and parity factors, and identify taxa and genes associated with reproductive success by artificial insemination.

**Results:**

The study identified *Staphylococcus*, *Escherichia*, and *Histophilus* as the most abundant genera. Microbial communities varied considerably between breeds and herds, with high predictive accuracy (> 90%) in classification models. Differential abundance analysis revealed that the genera *Histophilus, Fusobacterium, Bacteroides, Campylobacter, Streptobacillus, Gemella, Peptoniphilus, Helococcus, Treponema, Tissierella*, and *Phocaeicola* were more abundant in non-pregnant ewes. Some of these taxa were also associated with four COG entries and one KEGG orthologue significantly linked to non-pregnancy, primarily involving carbohydrate metabolism, defence mechanisms, and structural resilience. Age and parity were also associated with microbiota composition, particularly in ewes older than five years or with more than three parturitions, suggesting that cumulative physiological changes may contribute to microbial shifts over time.

**Conclusions:**

The ewe’s vaginal microbiome appears to be mainly influenced by both herd and breed, though distinguishing genetic from environmental factors is challenging within our study design. While the overall microbiota showed a subtle effect on pregnancy, certain genera had a significant negative impact, likely due to pathogenic or inflammatory properties that disrupt reproductive health. The metagenomic approach used here enabled not only comprehensive taxonomic classification but also detailed functional analysis, providing deeper insights into the microbiome’s role in reproductive outcomes.

Video Abstract

**Supplementary Information:**

The online version contains supplementary material available at 10.1186/s40168-025-02165-z.

## Background

Ovine agriculture holds significant economic importance in Spain, ranking as the second-largest producer in Europe and the fifth worldwide. The profitability of the sector, which heavily depends on fertility rates, stands out as a major challenge [1]. The strategic use of Artificial Insemination (AI) has revolutionised breeding programmes in dairy ruminants, offering key advantages in progeny testing, herd connectivity, and the extensive dissemination of genetic improvement. Despite these advancements, AI success rates in sheep remain suboptimal, ranging from 30 to 60% [2–4]. This contrasts sharply with the higher success rates observed in other livestock species. For example, pregnancy rates after AI typically range from 50 to 75% in cattle [5, 6], 65 to 72% in dairy goats [7], and up to 90% in the pig industry [8]. Multiple factors contribute to the low fertility rates of AI in sheep, such as the unique morphology of the ewe reproductive tract, mandatory use of fresh semen, and the difficulty in identifying the precise phase of the ewe’s ovulatory cycle [9].

In the last decade, considerable research has indicated that the microbial composition of the reproductive system is critical for human fertility. Studies highlight the significant role of microbiota in fertility and assisted reproductive technologies, demonstrating that changes in the male or female reproductive microbiota can lead to reproductive disorders and may impact reproductive success, as well as connections with other microbial communities, such as those in the gut [10–17]. This recognised importance of microbiota in human reproductive health has laid the groundwork for similar research in animal farming, particularly with ruminants. In cattle, some investigations have characterised the reproductive microbiota [18], and other studies have shown that microbial communities can influence reproductive efficiency and overall health in bovines [19–22]. In sheep reproductive health there is limited research, and most studies focus on natural mating [23–25]. References associating microbiota and fertility by AI are scarce, with our research group being among the pioneers in addressing this issue [26, 27]. Under this context, recent studies by Serrano et al. [28], Koester et al. [23], Reinoso-Peláez et al. [27], and Barba et al. [24] have identified microbial taxa closely associated with sheep pregnancy outcome. Remarkably, genera such as *Neisseria, Oenococcus, Mageebacillus, Histophilus, Actinobacillus,* and *Sneathia* were found in greater abundance in non-pregnant ewes. Conversely, *Mannheimia, Oscillospiraceae,* and *Alistipes* were more prevalent in ewes that successfully achieved pregnancy, suggesting a beneficial impact of these genera on reproductive efficiency. Recent studies from our group by Serrano et al. [28] and Reinoso-Peláez et al. [27] observed that progesterone intravaginal releasing devices (PRIDs), a treatment for oestrus synchronisation commonly used to conduct AI in sheep, influence microbiota composition. Greenwood et al. [29], using 16S ribosomal ribonucleic acid (rRNA) gene sequencing, identified significant differences in the vaginal microbiome between different sheep breeds (Poll Merino and White Suffolk), as well as in production traits such as fleece and post-weaning weight, suggesting that both environmental and genetic factors may be involved in microbiota composition.

The study of the composition of microbiota has been facilitated by the advances in high-throughput sequencing technologies. Metagenomics has particularly emerged as the preferred approach for analysing the microbiome. The amplification and sequencing of the 16S rRNA gene have become molecular fingerprints for microbial taxonomy, acting as a genetic identifier for species [30]. Nonetheless, advanced techniques like third-generation sequencing are revolutionising the field by significantly enhancing our understanding of the microbiome and providing a deeper perspective of its functional role in the homeostasis of the host organism. Nanopore sequencing, a technique pioneered by Oxford Nanopore Technologies (ONT), monitors changes in electrical currents as nucleic acids pass through a protein nanopore. This procedure not only allows classifying organisms based on sequence similarity but also elucidates gene functions and their interactions, with the aid of databases such as COG (Clusters of Orthologous Groups) and KEGG (Kyoto Encyclopedia of Genes and Genomes). The generation of longer reads significantly enhances the robustness of taxonomic assignment, improves annotation accuracy, and facilitates the identification of a broader range of taxa and functional pathways. These advantages allow researchers to move beyond purely taxonomic analyses, offering deeper insights into microbial roles in health and disease, as well as potential pathogenic or protective mechanisms. Although this technique has its limitations, such as database selection and challenges associated with high concentrations of host DNA [27, 31–34], it is in continuous evolution, with constant advancements improving its accuracy and reliability (e.g. implementing adaptive sequencing). In this line, while previous studies in sheep have predominantly relied on 16S rRNA amplicon sequencing or culture-based methods, studies employing a long-read metagenomic approach in sheep fertility remain absent. The only available reference on long-read metagenomics in sheep reproductive microbiota is our own pilot study [27]. However, that study was primarily focused on assessing the effect of intravaginal sponges rather than establishing broader microbiota-fertility associations. This underscores the need for more robust studies employing novel metagenomic approaches to provide a more comprehensive understanding of microbial roles in fertility by AI.

The current study aims to investigate the interplay between the ovine vaginal metagenome and its genes with fertility by AI and other factors under study. The specific objectives include: (i) to describe the microbial composition content of the sheep vaginal reproductive tract; (ii) to identify the extent to which the effects of breed, herd, age, and parity are associated with the composition and abundance of the microbiota, through beta diversity analysis and a predictive approach based on microbiota composition as a proxy; (iii) to determine the potential association between the composition and abundance of microbial communities and sheep pregnancy outcome by AI; and (iv) to examine the function of the genes identified and their interactions with the different factors under study. To achieve this goal, vaginal samples from 297 ewes from three Spanish breeds, belonging to four different flocks, were analysed using nanopore metagenome sequencing. This study complements and extends the scope of analogous research conducted by our team using the same animals with a 16S rRNA metabarcoding approach [35], enabling a thorough and extensive analysis in terms of both analytical types and sample size, for a more detailed understanding of the ovine vaginal microbiota.

## Material and methods

### Animal samples

Animal manipulations were performed according to the Spanish Policy for Animal Protection RD 53/2013, which meets the European Union Directive 2010/63/EU about the protection of animals used in experimentation.

**Fig. 1:**
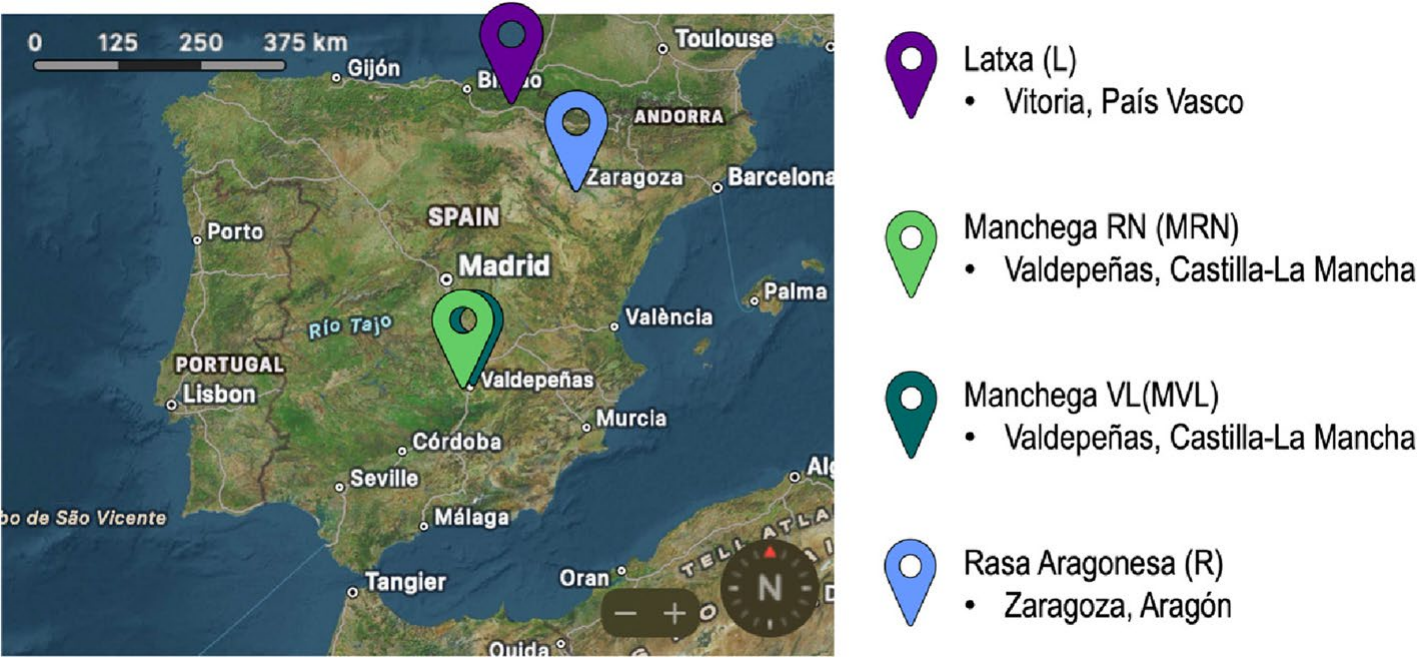
Geographic locations of the farms where the studied ewes were raised. Each coloured marker represents a different breed-herd: Latxa (L), Manchega RN (MRN), Manchega VL (MVL), and Rasa Aragonesa (R). The map follows a north-oriented standard projection, with north positioned at the top. The scale bar in the upper left corner provides a reference for distance, where each segment represents 125 km, with total markers at 0, 125, 250, and 375 km. This allows for an approximate estimation of distances between locations. The map was generated using Apple Maps (macOS)

The research involved 297 ewes, aged from 2 to 5 years, representing three different purpose breeds (Latxa, Rasa Aragonesa, and Manchega) reared in different geographic regions of Spain. Latxa is a dairy sheep breed, Rasa Aragonesa is a meat sheep breed, and Manchega is used for both milk and meat production. The sample consisted of 66 Latxa ewes (herd L), 97 Manchega ewes —of which 56 belonged to the Manchega RN (MRN) herd, a non-commercial herd managed by the breeders association of livestock farmers, and 41 to the Manchega VL (MVL) herd, a commercial operation— and 134 Rasa Aragonesa meat ewes (herd R). The overall mean age of the ewes was 4.21 (SD = 2.49) years, with herd-specific averages of 3.01 (SD = 1.63) years for L, 2.58 (SD = 0.94) years for MRN, 3.46 (SD = 1.41) years for MVL, and 5.71 (SD = 2.70) years for R. The mean parity was 3.48 (SD = 2.44), with herd-specific values of 2.91 (SD = 1.57) for L, 2.16 (SD = 1.13) for MRN, 3.28 (SD = 1.90) for MVL, and 4.38 (SD = 2.99) for R. Both the age and parity categories were divided into five groups to explore potential non-linear relationships. The age categories were as follows: < 2 years, 2–3 years, 3–4 years, 4–5 years, and > 5 years. For parity, the categories were < 2 parturitions, 2–3 parturitions, 3–4, 4–5, and > 5 parturitions. This was done to explore any potential non-linear relationships of these variables. Management systems varied by breed and production purpose. Latxa ewes were reared under semi-extensive conditions for milk production, with high pasture use, and a milder climate with higher humidity. Manchega ewes were managed intensively for dairy, with limited pasture access, trough feeding, and exposure to extreme seasonal temperatures and low humidity. Rasa Aragonesa ewes were raised under a semi-extensive meat production system, with greater pasture use, supplementary trough feeding, and exposure to a climate with higher humidity and extreme seasonal temperatures. The locations of the farms are shown in Fig. 1. All these animals had lambed at least once. All ewes were in good corporal condition, and no health event problems or infections were observed at the time of insemination. Inseminations were performed in the same season for all ewes within the same herd, spanning between August and September 2020.

All ewes underwent oestrus synchronisation using PRID devices containing 20 mg of Flurogestone acetate (Chronogest. MSD Animal Health, Kenilworth, NJ, USA). Only in the Latxa breed ewes (herd L), PRIDs included an antibiotic treatment by imposition of the farmer, which consisted of 0.6 g of powdered Framycetin (neomycin sulfate, Framicas, Laboratorios Ovejero, Spain). After 14 days, PRIDs were removed, and the effectiveness of the synchronisation was confirmed by progesterone testing on the day of AI. Subsequently, ewes received an injection of 300 to 500 IU of PMSG (pregnant mare’s serum gonadotropin) tailored to their body weight to stimulate ovulation. Artificial insemination was then performed 53 to 55 h following PRID removal. Prior to insemination, a vaginal exudate sample was collected from each ewe using a vaginal swab (Real Vaginal Microbiome DNA Kit, Durviz S.L., Valencia, Spain). Samples were kept on dry ice in an insulated container during collection and subsequently stored at − 80 °C in the laboratory until DNA extraction. To avoid cross contamination, a speculum was used during sample collection and was disinfected between ewes using a povidone-iodine solution. The inseminations utilised fresh semen from 13 Rasa Aragonesa, 12 Latxa, and 10 Manchega rams, aged between 4 and 7 years. As for the ewes, all rams were in good condition and no health events or infections were observed at the time of insemination and were managed under similar conditions in the AI centres. Minimum quality control for semen included mass motility above 3.5 (scored 0 to 5) and individual motility above 80/4 (scored 0–100/0–5). For Rasa Aragonesa and Manchega breeds, sperm doses were prepared using fresh semen at a concentration of 300–400 million spermatozoa/mL, diluted in INRA96® (IMV Technologies, L’Aigle, France), and supplemented with penicillin, gentamicin, and amphotericin B in 0.25 mL straws. For Latxa, sperm doses were prepared with powdered skimmed cow’s milk, streptomycin, penicillin, and sodium sulphanilamide in 0.25 mL.

Pregnancy was diagnosed via transabdominal ultrasound between 37 and 55 days post-insemination. Fertility results were determined from ultrasound findings adjusted for actual birthing outcomes: positive ultrasounds were classified as “positive” for pregnancy, while negative ultrasounds were categorised as “negative” unless a birth occurred. This approach aimed to evaluate fertilisation success and examine the association between pregnancy potential and vaginal microbial communities (Table 1).

**Table 1:** Fertility rate (number of animals) across herds, based on P4 levels, ultrasound, birth outcomes, and corrected pregnancy

Herd	P4	Ultrasound	Birth outcome	Corrected pregnancy
L	NA	83.08 (65)	75.76 (66)	81.82 (66)
R	56.91 (123)	46.2 (134)	42.54 (134)	46.27 (134)
MVL	60.98 (41)	26.83 (41)	29.27 (41)	34.15 (41)
MRN	32.73 (55)	23.64 (55)	21.43 (56)	25 (56)
Total	51.60 (219)	47.46 (295)	44.11 (297)	48.49 (297)

*P4* pregnancy diagnosis based on progesterone levels, which was not performed in Latxa (L) and therefore not considered in the analysis. *Ultrasound* pregnancy diagnosis via ultrasound. *Birth outcome* successful delivery. *Corrected pregnancy *ultrasound results corrected by birth outcome. If ultrasound is positive, the value is considered positive; if ultrasound is negative but birth outcome is positive, the value is also considered positive; and if ultrasound is positive but birth outcome is negative, the value remains positive. This approach aims to assess the conception effect of microbial communities while minimising environmental biases such as stress-induced abortions or other external pathological factors. All values are expressed as percentages, with the total number of animals used to estimate the percentage shown in parentheses

*L* Latxa, *MRN* Manchega RN, *MVL* Manchega VL, *R* Rasa

### DNA extraction and sequencing

Swabs saturated with vaginal secretion were trimmed and inserted in individual Eppendorf tubes. Real Vaginal Microbiome DNA Kit, (Durviz S.L., Valencia, Spain) was used following the protocol instructions. DNA samples were eluted in 15 to 30 µl DEPC water. Genomic DNA concentration, and quality ratios 260/280 and 260/230 were measured using a Qubit 4 fluorometer (Thermo Fisher Scientific, DE, USA) with the Qubit ™ ds DNA BR Assay Kit, and a Nanodrop 2000 spectrophotometer (Thermo Fisher Scientific, DE, USA), respectively.

Microbial DNA sequencing was carried out using nanopore technology with a GridION sequencer (Oxford Nanopore Technologies, ONT). For each sequencing run, 400 ng of DNA in an 11 µl volume was prepared according to the Ligation Sequencing gDNA Native Barcoding Kit 24 V14 (SQK-NBD114.24) protocol. Samples were multiplexed in 13 runs, each of them employing 24 different barcodes, including corresponding negative controls. The barcoded samples were pooled in a 1.5-mL Eppendorf DNA LoBind tube to perform adapter ligation for sequencing using R10.4.1 flow cells. Samples were sequenced using the adaptive sampling method to reject sequences that map against the Oar_rambouillet_v1.0 (GCA_002742125.1) sheep reference genome.

### Bioinformatic analysis

The fast5 sequences generated were processed through the basecalling procedure using Guppy software (version 6.4.6) provided by ONT. The Super Accuracy basecalling algorithm was used [36] which guarantees a sequence minimum quality of 10 (Q10).

Following basecalling, barcode trimming was performed. Subsequently, reads shorter than 200 bp were discarded. Sequencing performance and quality were assessed using the NanoPlot software. Given the considerable amount of host DNA sequenced, despite the adaptive sampling strategy applied, a post-sequencing filtering step was implemented. For this purpose, the Minimap2 software [37] was used to obtain a long-read alignment against the Oar_rambouillet_v1.0 (GCA_002742125.1) *Ovis aries* reference genome from NCBI. All sequences that aligned to the sheep reference genome were systematically discarded, retaining only those sequences that did not align.

After that, we obtained a total of 48.16 Gb corresponding to 63,430,285 reads, with an average read length per sample of 759.3 bp and an average number of reads per sample of 213,570 bp with SD of 142,223 and a median of 186,751. The per-sample read count ranged from 8507 to 1,149,191 with an interquartile range of 163,008. Retained reads were analysed using the SqueezeMeta v1.6.2 pipeline for long reads [38], which performs Diamond Blastx against NCBI-nr, COG, and KEGG databases. SqueezeMeta implements a lowest common ancestor (LCA) algorithm to find the consensus taxon for each read. This pipeline also aligns each read to a gene reference database and provides the number of copies of each gene present in the sample. Gene functions were annotated using the best hit above a minimum score threshold of 60 (genus), 55 (family), 50 (order), 46 (class), and 42 (phylum), which are the default values of SqueezeMeta software. Hits below these thresholds were considered unclassified taxa.

To streamline the execution of SqueezeMeta, we implemented a custom workflow in Bash and Snakemake that managed job submission and execution within the SLURM scheduling system. The process followed these steps: (i) filtered sequence reads were processed individually for each of the 297 samples. (ii) A dedicated script was automatically generated for each sample, executing sqm_longreads.pl from SqueezeMeta with default parameters and the -euk flag to enable eukaryotic classification. (iii) These scripts were submitted to SLURM, which scheduled and executed them in parallel, optimising computational resources and processing time. (iv) The system continuously monitored job status, dynamically submitting pending analyses while respecting queue limits. The computational script and workflow associated with the SqueezeMeta process can be found in the Supplementary material 1.

All bioinformatics processes were computationally intensive and time-consuming, requiring substantial computing power. To overcome these challenges, we leveraged the supercomputing infrastructure at CESGA (Centro de Supercomputación de Galicia, www.cesga.es), allowing us to efficiently process large-scale metagenomic data.

### Microbiota composition and diversity analysis

The results obtained from the SqueezeMeta pipeline were filtered to retain reads that mapped to microorganisms belonging to Bacteria and Archaea domains, as well as sequences corresponding to viral entities and phyla within the Protista (Apicomplexa, Euglenozoa, Evosea), Fungi (Ascomycota), and Animalia (Nematoda) kingdoms.

Three variables were considered for the analyses: (i) breed, with three levels: Latxa, Manchega, and Rasa Aragonesa; (ii) herd, with four levels: two Manchega herds (MVL and MRN), one Latxa herd, and one Rasa Aragonesa herd; and (iii) pregnancy status, with two levels: positive and negative. The male effect was not considered, as microbiota samples were collected prior to AI, so the male has no influence on the vaginal microbiota assessed in the ewes.

To determine the microbial composition, taxa were grouped at the genus and phylum levels. The core microbiota was defined as those taxa that were present in at least 90% of the samples. This threshold was selected to ensure a high level of representation across samples, following precedents in microbial ecology where a balance of inclusivity and specificity is critical [39–41]. Relative abundances (RA) were used to visualise and interpret the composition of the core microbiota.

Alpha diversity, the microbiota diversity within a single sample, was calculated using four indices: (i) observed, which counts the number of unique species present, (ii) Chao1, which estimates the species richness taking into account rare species, (iii) Shannon, defined as the number of species in a community weighted by their abundance and evenness of distribution, and (iv) InvSimpson, which quantifies the probability that two individuals randomly selected from a sample belong to the same species [42]. Rarefaction was performed using a threshold of 6000 read counts (the majority of the curves reach a plateau on this threshold, Supplementary material 2: Fig. S5), and statistical significance was assessed using the Wilcoxon rank-sum test.

Beta diversity, the dissimilarity between samples based on microbial communities, was estimated using principal component analysis (PCA) and PERMANOVA to evaluate differences associated with herd, breed, age category, parity category, and pregnancy. PERMANOVA was implemented under a global model to consider all groups from the variable of study, as well as in pairwise comparisons between groups. A total of 999 permutations were implemented for both the global analysis and the pairwise comparisons. Pregnancy-related differences were assessed both for all samples collectively and within each herd. Here, the dataset was normalised using the CLR transformation to address its compositional nature, with the microbiome R package [43]. Principal component analysis was conducted using the prcomp function from the Stats R package [44]; PERMANOVA was performed with the vegan [45] and pairwise comparisons were conducted using the RVAideMemoire [46] R packages.

A random forest (RF) model was implemented to evaluate the predictive capacity of the vaginal microbiota for pregnancy, herd, breed, age, and parity. The accuracy of the model was assessed by cross-validation (CV) using a k-fold approach. The model was established as follows:

$$y= \mu + \sum_{t=1}^{T}{c}_{t}{h}_{t}({y}_{n};{M}_{m,t})$$

where $y$ is the expected phenotype (pregnancy, herd, breed, age, or parity; a separate model was constructed for each variable), $\mu$ correspond to the mean; $T$ is the number of trees selected in the forest (*T* = 10,000), where each tree is built through bootstrap aggregation; ${c}_{t}$ is a shrinkage factor that estimates the average of the regression trees; ${h}_{t}({y}_{n};{M}_{m,t})$ is a random tree; where $M$ represents the abundance of the microbiota in CLR composition (at the phylum, genus, COG, or KEGG levels); each tree was constructed using a random sample of $n$ = 63.3% (two-thirds of the dataset); $m$ is the number of randomly selected variables (taxa, COG or KEGG) at each node split, $m= \sqrt{p}$, where $p$ is the total number of predictor variables. The model's loss function is based on the out-of-bag (OOB) samples, which are used to measure the prediction error. For model training and more robust results, 5-fold CV was performed as follows: The dataset was randomly partitioned into five subsets of equal size. In each fold, four subsets were used for training and the remaining subset was used for testing. The performance of the RF model was assessed by calculating the overall classification accuracy for categorical responses (pregnancy, herd, breed, age category, or parity category), while Pearson correlation (*r*) and root mean squared error (RMSE) were used for continuous outcomes (age and parity as numerical factors). A prevalence filter of 1% was applied to exclude very rare taxa or genes, and CLR transformation was applied to the data for compositional normalisation. The randomForest R package was used to perform the RF analysis [47], while base R was utilised for conducting the K-fold CV [44].

Finally, differential abundance analysis was performed to identify variations in taxa and genes associated with pregnancy across different levels, including genus, phylum, COG and KEGG using the DESeq2 R package [48] and applying a prevalence filter of 1%. This package employs negative binomial generalised linear models and calculates size factors for data normalisation. Additionally, after testing for different methods, the poscount method was used, which calculates a modified geometric mean by taking the n-th root of the product of non-zero counts. This approach is particularly useful for datasets with many zeros and was developed in part for metagenomic studies, including collaborations with Paul McMurdie’s phyloseq package. Two approaches were carried out to implement the differential abundance analysis for pregnancy, to which we will refer as (i) global model, when all samples were considered for the analysis, and (ii) herd-specific model, when a within-herd analysis was performed. To avoid overparameterisation and given that the variables breed and herd only differ in one additional level (in the case of herds referred to Manchega breed), as well the variable age and parity displayed a high correlation (*r* = 0.94) the primary variables considered for the model were pregnancy, herd, and age category. This decision was validated through the significance of the PERMANOVA results. The models were represented as

$${\varvec{y}}={\varvec{X}}{\varvec{b}}+{\varvec{e}}$$

where $y$ is the vector of abundance of a given taxon, COG or KEGG orthologue; *b* is a vector that includes the fixed effects of pregnancy, herd, and age category, in the case of the global model, and pregnancy and age category in the case of the herd-specific model; *e* is the vector of residual errors and *X* is incidence matrix for the fixed effects. False discovery rate (FDR) multitest correction was applied to adjust p-values at 5% level.

Reads assigned to specific COG and KEGG entries identified as differentially expressed in relation to pregnancy, were mapped to their corresponding taxa. Specifically, for each sequence, we recorded both the assigned genus (or phylum) and the assigned functional annotation (COG or KEGG). The analysis prioritised the 15 most abundant genera and the 5 most abundant phyla to illustrate which taxa carry those differentially abundant genes and to identify those taxa that were also differentially abundant in pregnancy contexts. Subsequently, network analysis was performed using the igraph [49] and ggraph [50] packages in R to visualise the relationships between differentially abundant genes and their associated pathways. Each gene was linked to its corresponding metabolic or functional pathway, constituting a bipartite network where nodes represented either genes or pathways, and edges denoted their associations.

## Results

The fertility measure selected for this study was "corrected pregnancy" (ultrasound outcome corrected by birth outcome; hereafter referred to simply as "pregnancy", Table 1). Average fertility rate for all ewes by AI was 48.49%. Within-herd fertility was 81.82% for Latxa, 46.27% for Rasa Aragonesa, 34.15% for Manchega VL, and 25% for Manchega RN.

### Microbiota composition and diversity analysis

The sequencing data from the 297 samples included a total of 63,430,285 reads. After quality filtering, 3,122,826 reads mapped to Bacteria, Archaea, Protista, and Fungi kingdoms, as well as to viruses. Taxonomic annotations included 113 phyla, 551 families, 1389 genera, 9911 COG entries, and 8417 KEGG orthologues. Table 2 shows summary statistics of read abundance on the microbiota composition.

**Table 2:** Summary statistics of reads abundance across taxonomic and functional levels

Level	Total reads	Mean sample	Median sample	Number of taxa/genes
Phylum	3,161,540	10,644.92	6250	113
Class	2,890,511	9732.36	5577	149
Order	2,497,095	8407.73	4338	284
Family	2,244,702	7557.92	3839	551
Genus	1,821,558	6133.19	3328	1,389
COG	3,662,878	12,332.92	6467	9,911
KEGG	3,776,544	12,715.64	6872	8,417

*Level* taxonomic or functional classification level, *Mean sample* the average number of reads per sample, *Median sample:* the median number of reads per sample, *Number of taxa/genes* the total number of distinct taxa or genes identified at each classification level. All the unclassified reads were discarded at each taxonomic level, retaining taxa that belong to the kingdoms bacteria, archaea, and viruses, as well as to the phyla apicomplexa, ascomycota, euglenozoa, evosea, and nematoda

**Fig. 2:**
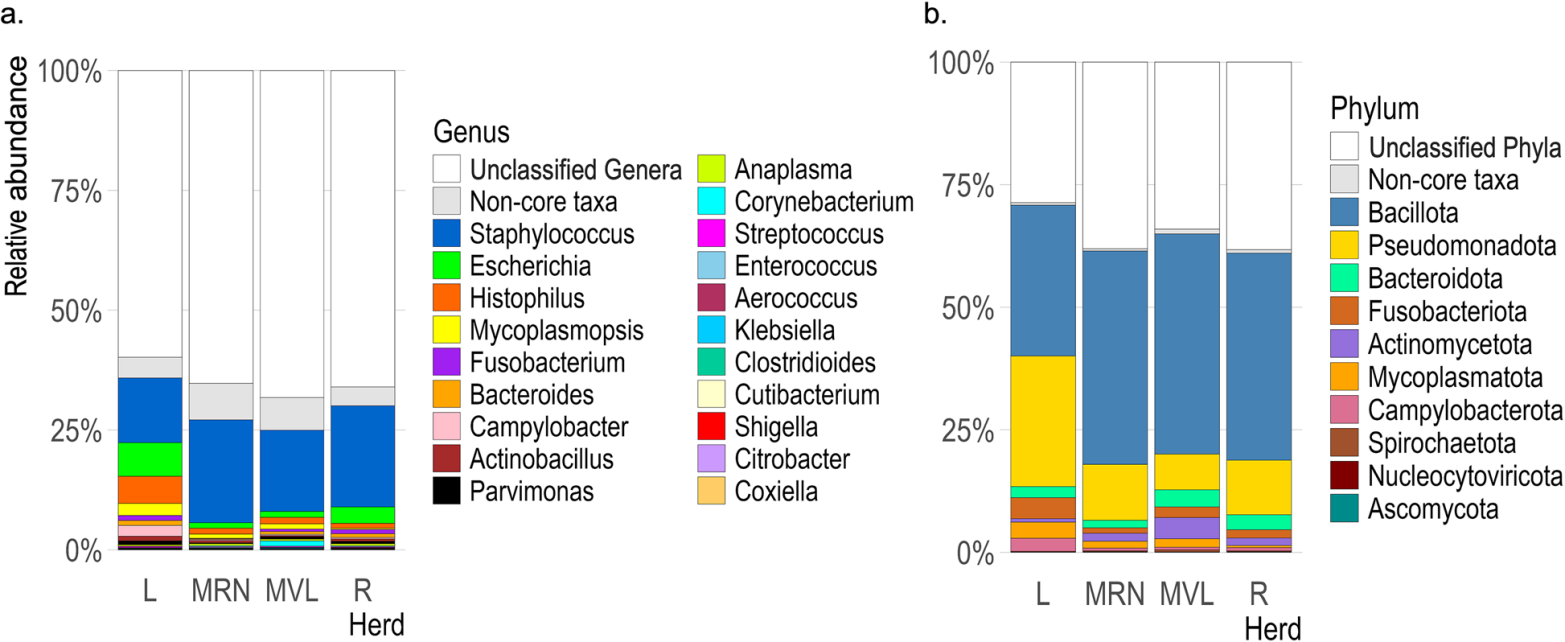
Microbial community composition at genus (**a**) and phylum (**b**) level. Taxa with a prevalence ≥ 90% were considered part of the core microbiota. L: Latxa, MRN: Manchega RN, MVL: Manchega VL, R: Rasa. KO: KEGG Orthologous. Non-core taxa: those taxa with a prevalence < 90%

The core microbiota comprised 11 phyla and 21 genera (Fig. 2). The most abundant genera included *Staphylococcus* (18%), *Escherichia* (3.2%), *Histophilus* (2.3%), *Mycoplasma* (1.2%), *Campylobacter* (0.7%), *Bacteroides* (0.7%), *Fusobacterium* (0.6%), *Actinobacillus* (0.5%), *Parvimonas* (0.5%), and *Anaplasma* (0.3%). Variations in these genera were observed among herd groups; *Staphylococcus* was more abundant in Rasa Aragonesa and Manchega RN (*p* = 4.715e− 16; Kruskal–Wallis test), whereas Latxa exhibited higher levels of *Histophilus* compared with other herds (*p* = 2.2e− 16; Kruskal–Wallis test). At the phylum level, Bacillota (64%), Pseudomonadota (20%), Bacteroidota (3.6%), Fusobacteriota (3.2%), and Actinomycetota (3%) were the most abundant.

**Fig. 3:**
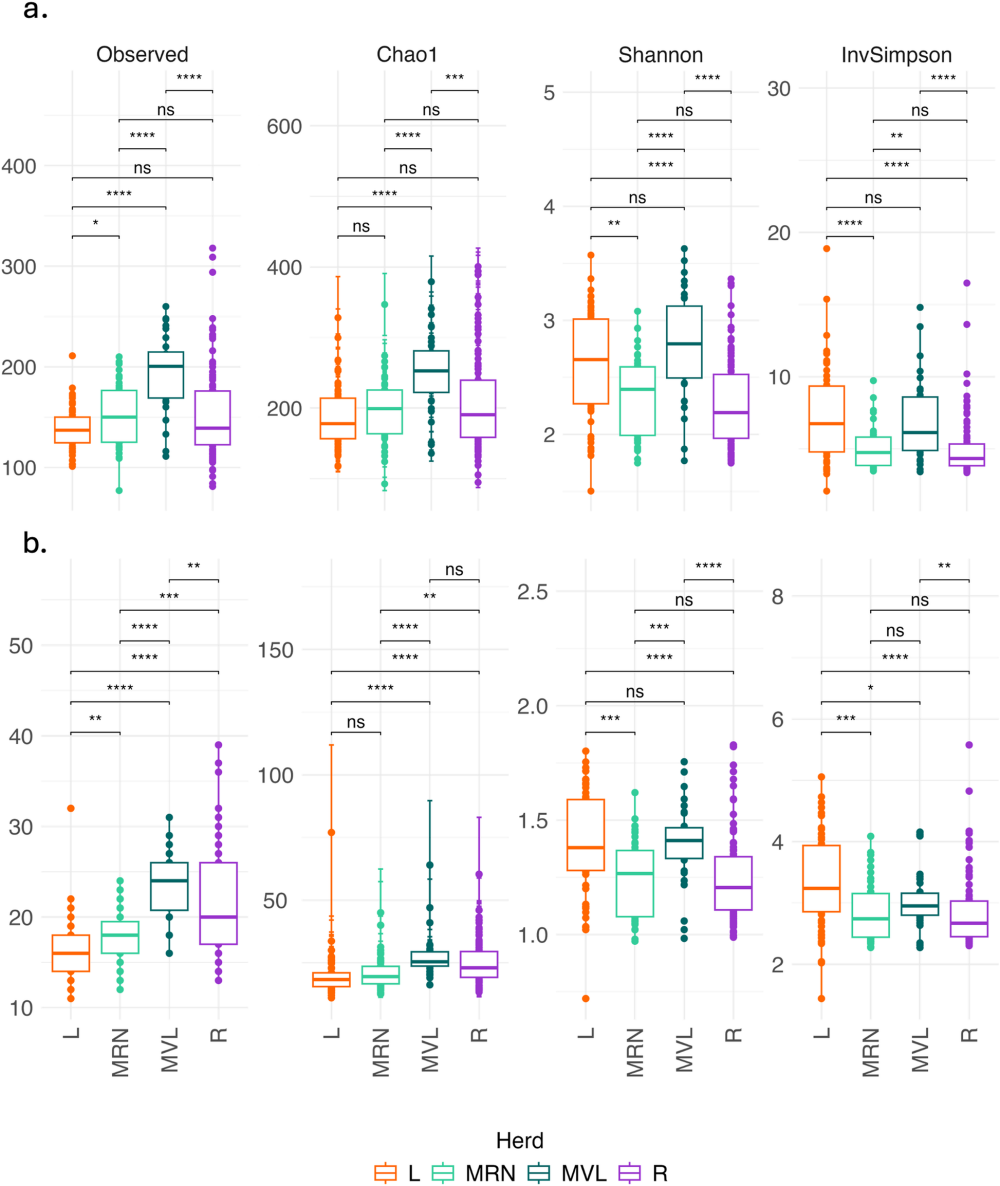
Alpha diversity measures for herd at the genus (**a**) and phylum (**b**) level. Four measures were considered (Observed, Chao1, Shannon, and InvSimpson index). Data were rarefied. Statistical significance is indicated by asterisks: * (*p* < 0.05), **(*p* < 0.01), *** (*p* < 0.001), **** (*p* < 0.0001) and “ns”: not significant

In general, alpha diversity exhibited significant variations among herds at phylum and genus levels (Fig. 3). Manchega VL presented the highest diversity across all indices. In contrast, Latxa displayed the lowest diversity in the Observed and Chao1 indices. However, for the Shannon index, the Latxa herd showed higher diversity, whereas the Rasa herd exhibited the lowest. No significant differences were observed between positive and negative pregnancy ewes for the levels analysed or across the entire sample set (Supplementary material 2: Figs. S1 and S2). Regarding age and parity categories, differences in alpha diversity were subtle. In general, ewes over 5 years old and those with more than 5 parturitions exhibited lower alpha diversity compared to some younger groups (e.g. < 2 or 2–3 years/parturitions), as shown in Supplementary material 2: Figs. S3 and S4.

**Fig. 4:**
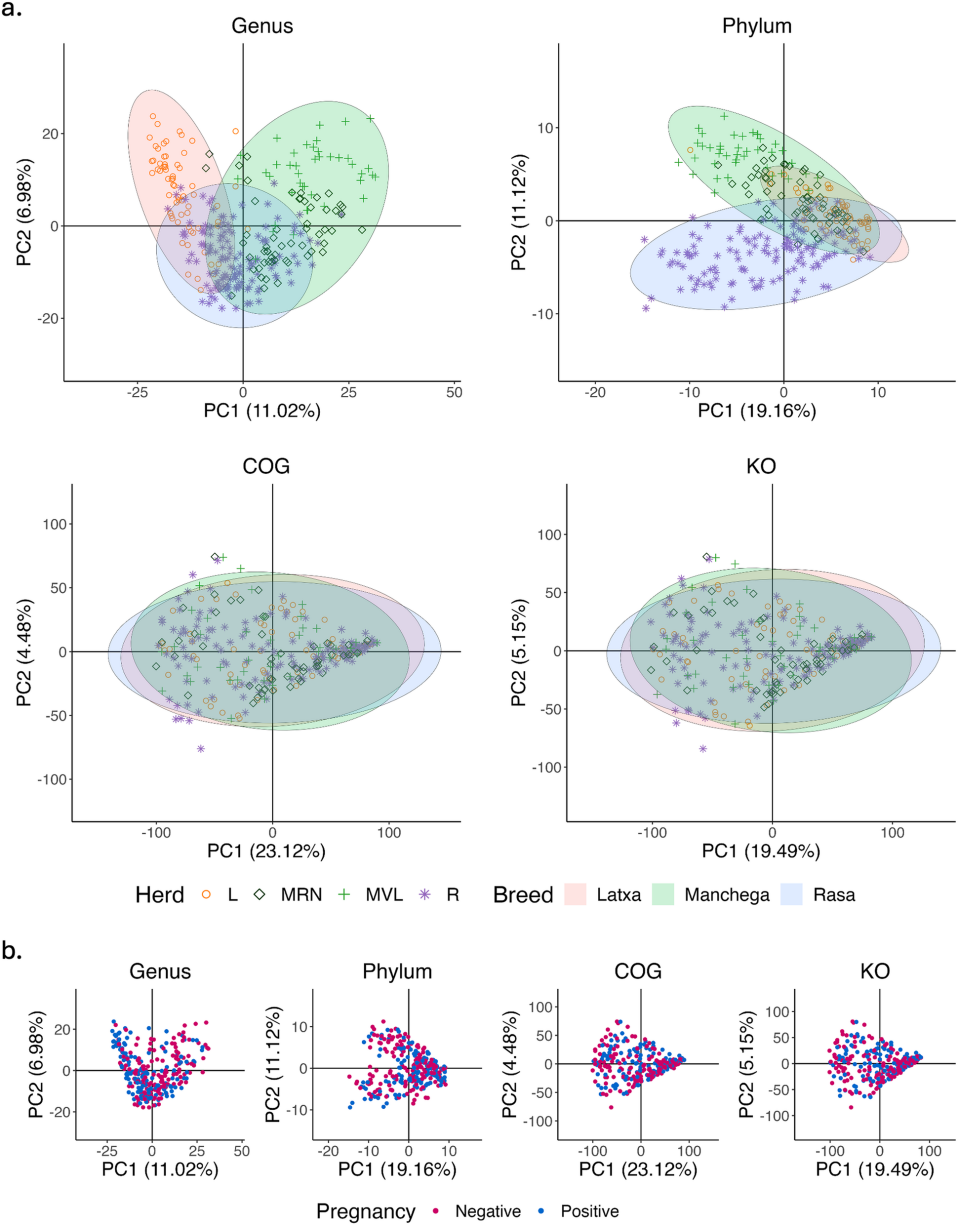
Principal component analysis of microbiota composition by breed, herd, and pregnancy status. Phylum, genus, COG, and KO (KEGG Orthologs) levels were analysed. Panel **a** shows the analysis for breed and herd, while panel **b** shows the PCA for pregnancy. Ellipses in panel (**a**) were calculated using covariance to represent data variability within each breed group

Principal component analysis revealed differences among breeds and herds at the genus level, with some degree of overlapping between Rasa Aragonesa and the other groups (although Rasa presented the highest number of animals). At the phylum level, the PCA separated to some extent Rasa Aragonesa and Manchega, while Latxa overlapped with the formers (Fig. 4a). Both herds of Manchega are grouped closely yet display distinct clusters. No differences were observed among groups when considering functional classification (COG and KEGG), or across pregnancy, age, and parity categories (see Fig. 4b for pregnancy, and Supplementary material 2: Fig. S6 for age and parity categories).

Results from PERMANOVA showed significant differences for breed and herd in the global comparison, with the differences between breeds (*F* = 21.52 at phylum and 20.44 at genus level) being slightly higher than those observed between the herds (*F* = 18.42 at phylum and 18.31 at genus level). These differences were also evident in all pairwise comparisons (supplementary material 2: Table S2). Whereas significant differences were observed for pregnancy status under the global model, no differences were observed under the within-herd model. Similarly, while significant differences were observed in the global model for age and parity categories, these differences were only evident within the R herd. Pairwise comparisons revealed variations in group comparisons, following a clear pattern: animals over five years differed significantly from all other age groups, and those with more than three parturitions differed from those with fewer. Global PERMANOVA results are in Table 3, while within-herd analyses and pairwise comparisons are in Supplementary material 2: Tables S1–S4.

**Table 3:** PERMANOVA results for the effects of herd, breed, and pregnancy on microbiota and gene composition

Level	Factor	SumOfSqs	*R2*	*F*	Pr(> *F*)
Phylum	Herd	8092.83	0.159	18.420	0.001
	Breed	6513.67	0.128	21.522	0.001
	Pregnancy	424.10	0.008	2.474	0.008
	Age category	1303.59	0.028	2.092	0.001
	Parity category	1302.72	0.028	2.090	0.001
Genus	Herd	67,636.83	0.158	18.312	0.001
	Breed	52,296.28	0.122	20.442	0.001
	Pregnancy	4394.10	0.010	3.057	0.001
	Age category	18,897.07	0.031	2.352	0.001
	Parity category	16,006.77	0.026	1.983	0.001
COG	Herd	264,896.48	0.071	7.403	0.001
	Breed	229,012.52	0.061	9.535	0.001
	Pregnancy	15,110.55	0.004	1.190	0.184
	Age category	82,987.99	0.022	1.648	0.008
	Parity category	72,909.75	0.019	1.444	0.020
KEGG	Herd	285,831.10	0.068	7.082	0.001
	Breed	242,043.93	0.057	8.927	0.001
	Pregnancy	19,077.24	0.005	1.337	0.101
	Age category	87,442.57	0.021	1.542	0.005
	Parity category	78,401.38	0.019	1.379	0.020

Various taxonomic (phylum, genus) and functional (COG, KEGG) levels were considered. SumOfSqs sum of squares, a measure of variance. R2: Coefficient of determination, indicating the proportion of variance explained by the factor. F F-statistic, a ratio used to determine the significance of the factor. Pr(> F) *P*-value, indicating the statistical significance of the factor. Values in bold indicate significant results (*P* < 0.05)

Random forest model demonstrated varying accuracies across factors and levels. For categorical variables, breed exhibited the highest accuracy, with 89.24% (SD = 5.88) at the phylum and 92.40% (SD = 3.29) at the genus levels. Herd also showed high accuracy, with 84.18% (SD = 6.45) at the phylum and 91.07% (SD = 3.64) at the genus levels, followed by pregnancy with 52.18% (SD = 10.57) at the phylum and 56.11% (SD = 4.73) at the genus levels, age category with 38.78% (SD = 5.84) at the phylum and 39.08% (SD = 5.29) at the genus levels, and parity category with 42.76% (SD = 4.26) at the phylum and 42.88% (SD = 5.45) at the genus levels. Accuracy at COG and KEGG levels was generally lower. For age and parity as numeric factors, the correlation was higher for age at the genus level (*r* = 0.47, SD = 0.11) compared to parity (*r* = 0.34, SD = 0.15). Results for RMSE were similar for age (RMSE = 2.07, SD = 1.64) and parity (RMSE = 2.09, SD = 1.50). All results are shown in Table 4.

**Table 4:** Prediction accuracy results (and their SD) from cross validation analysis using a random forest algorithm

	Phylum	Genus	COG	KEGG
% Correctly classified				
Breed	89.24 (5.88)	92.40 (3.29)	70.20 (3.01)	70.11 (3.40)
Herd	84.18 (6.45)	91.07 (3.64)	67.57 (5.75)	69.27 (6.11)
Pregnancy	52.18 (10.57)	56.11 (4.73)	56.30 (6.30)	55.48 (6.05)
Age category	38.78 (5.84)	39.08 (5.29)	31.99 (9.55)	32.58 (7.50)
Parity category	42.76 (4.26)	42.88 (5.45)	40.12 (10.47)	39.19 (9.89)
Correlation				
Age	0.43 (0.06)	0.47 (0.11)	0.32 (0.07)	0.31 (0.05)
Parity	0.23 (0.03)	0.34 (0.15)	0.19 (0.10)	0.18 (0.11)
RMSE				
Age	2.02 (1.71)	2.07 (1.64)	1.88 (1.37)	1.91 (1.39)
Parity	2.08 (1.58)	2.09 (1.50)	1.75 (1.39)	1.77 (1.37)

For categorical variables, including Herd, Breed, Age category, Parity category, and Pregnancy, accuracy is measured as the percentage of correctly classified instances, which reflects the proportion of predictions where the model’s output matches the real category labels. For numerical variables, such as Age and Parity, accuracy is assessed using the correlation coefficient, which quantifies the linear relationship between predicted values and real values. The values in parentheses represent the standard deviation (SD) of the predictive accuracy across the cross-validation folds (*K* = 5)

### Differential abundance analysis

**Fig. 5:**
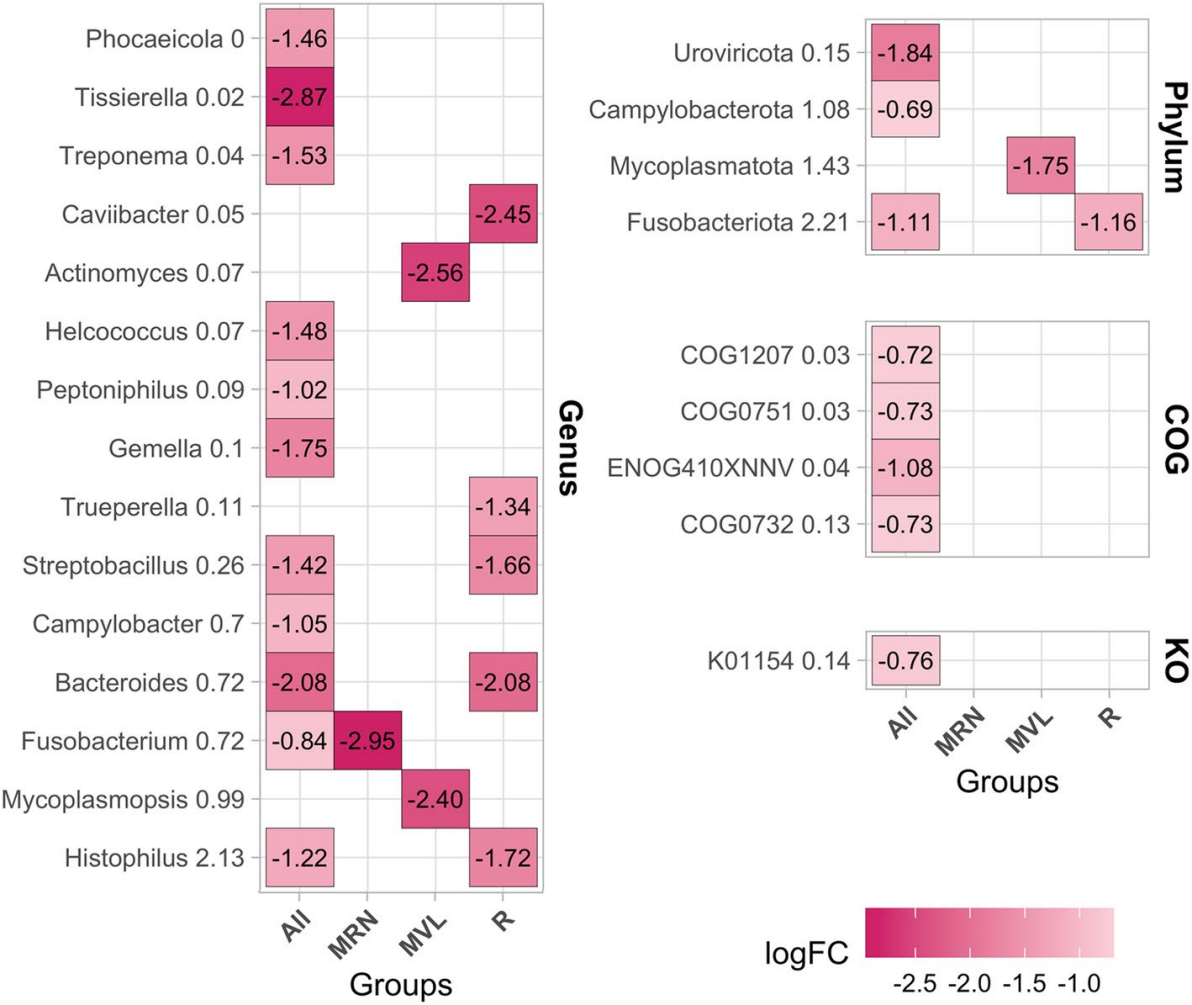
Heatmap showing significant results (FDR at 5%) for differential abundance analysis for pregnancy success at genus, phylum, COG, and KO (KEGG Orthologous) levels. The x-axis represents results for the global model (ALL) as well as within the Manchega RN (MRN), Manchega VL (MVL), and Rasa (R) herds. No significant taxa were associated with the Latxa (L) herd. The log fold change (FC) is represented by colour gradations and its corresponding value, with red indicating higher abundance in non-pregnant ewes and blue indicating higher abundance in pregnant ewes. The colour intensity correlates with the logFC value; values close to zero are represented in white. The logFC value is also presented in each square of the heatmap. The *y*-axis categorises the taxonomic assignments followed by its RA

Figure 5 shows the results of the differential abundance analysis for pregnancy success at the genus and phylum levels, as well as for the COG and KEGG categories under the global model, Manchega RN, Manchega VL, and Rasa. No significant taxa were found for the Latxa herd. Under the global model, 11 genera were significantly more abundant in non-pregnant ewes, including *Histophilus, Fusobacterium, Bacteroides, Campylobacter, Streptobacillus, Gemella, Peptoniphilus, Helococcus, Treponema, Tissierella,* and *Phocaeicola.* At the phylum level, Fusobacteriota, Campylobacterota, and Uroviricota were more abundant in non-pregnant ewes. Additionally, four COG entries and one KEGG orthologue were significantly more abundant in non-pregnant ewes.

When performing the analysis within individual herds, differences were observed. For instance, in Manchega RN, the genus *Fusobacterium* was notably more abundant in non-pregnant ewes. In Manchega VL, the genera *Mycoplasmopsis* and *Actinomyces,* and the phylum Mycoplasmatota were significantly more abundant in non-pregnant ewes. A similar trend was found in Rasa Aragonesa, where the genera *Histophilus*, *Bacteroides*, *Streptobacillus*, *Trueperella*, and *Caviibacter*, and the phylum Fusobacteriota were significantly more abundant in non-pregnant ewes. The RA and prevalence of these taxa and functional pathways are shown in Supplementary material 2: Figs. S7 and S8, and their distribution is illustrated through supplementary boxplots in Figs. S9–S15.

**Fig. 6:**
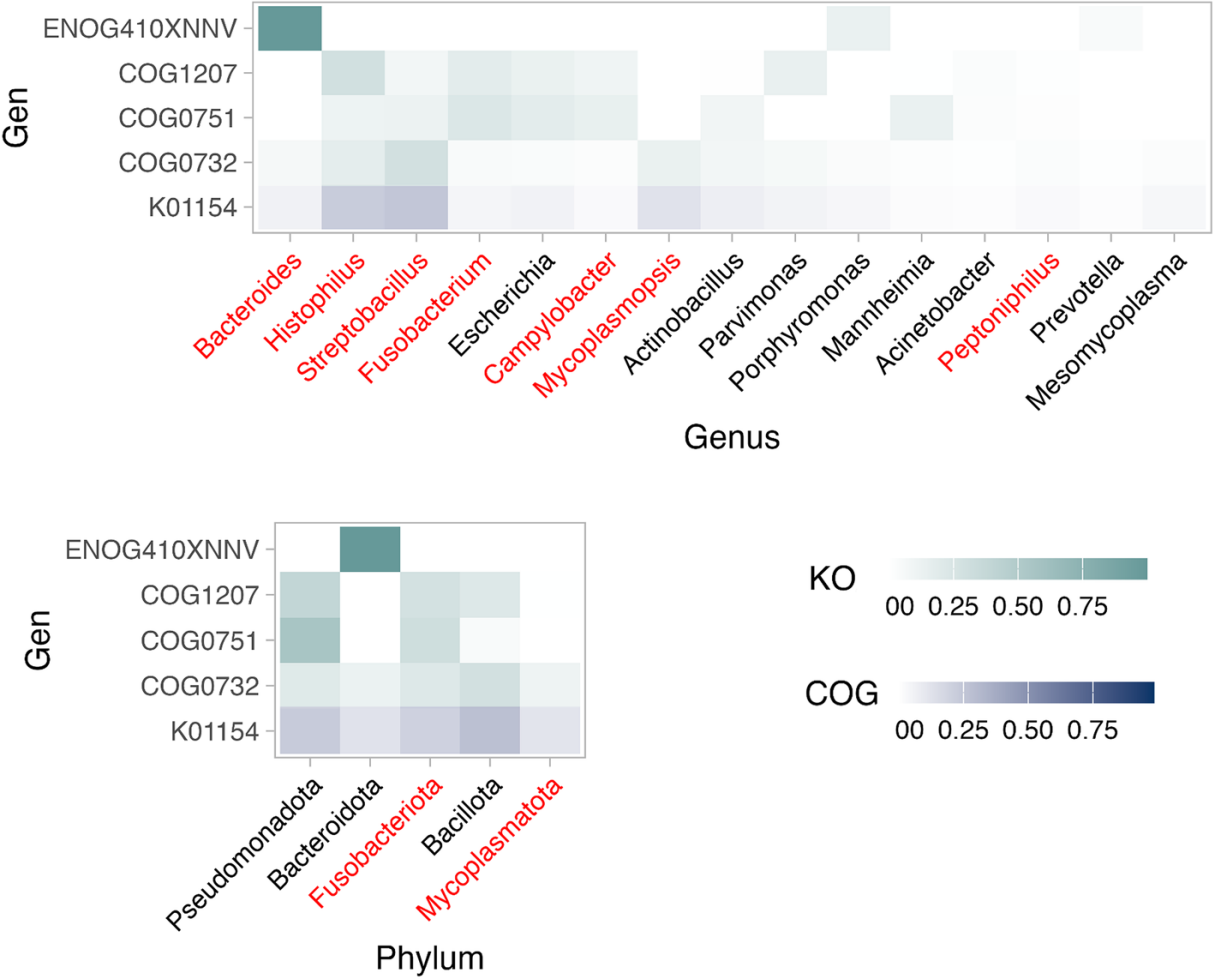
Heatmap of the relationship between genes and taxa differentially expressed in relation to pregnancy. The *y*-axis displays the genes (COG in green and KEGG in blue) that are significantly associated with pregnancy status, along with their corresponding taxa represented in terms of relative abundance (RA). The *x*-axis outlines the 15 most abundant genera (**a**) and the 5 most abundant phyla (**b**) associated with these COG and KO (KEGG Orthologous). Taxa that were significantly more abundant in non-pregnant ewes are highlighted in red, while those with no significant association are shown in black

Sequences aligned with the more significant abundant COG entries and KEGG orthologs in non-pregnant ewes corresponded predominantly to *Bacteroides*, *Histophilus*, *Streptobacillus*, *Fusobacterium*, *Campylobacter*, *Mycoplasmopsis*, and *Peptoniphilus*, as well as to the phyla Fusobacteriota and Mycoplasmatota. Figure 6 shows a heatmap based on these read co-assignments, illustrating which taxa harbour specific COG or KEGG functions linked to non-pregnancy.

**Fig. 7:**
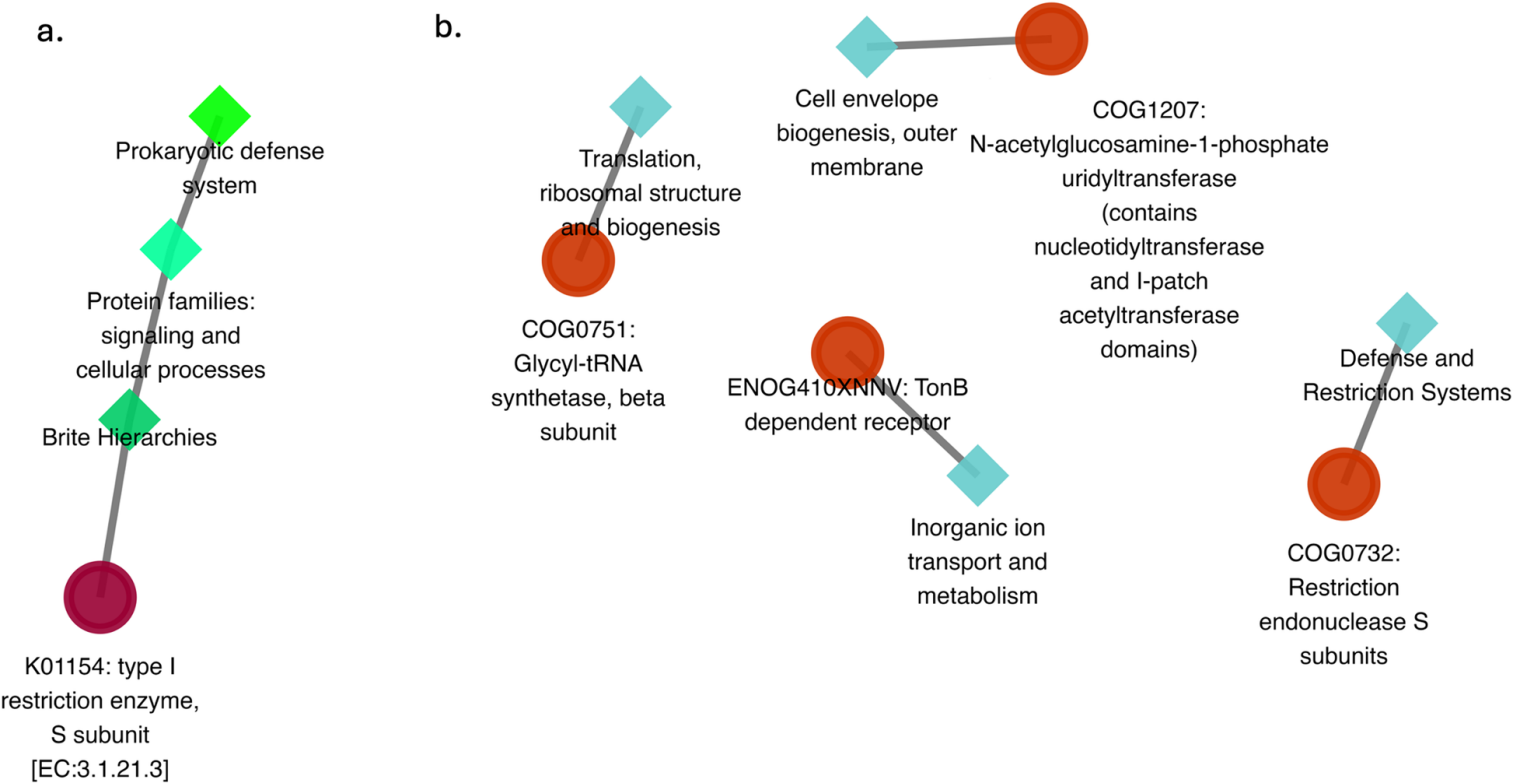
Network diagram of pathways associated with genes differentially expressed in pregnancy. Panel (**a**) displays the pathways involving KEGG orthologs, while panel (**b**) illustrates the pathways linked to COG entries

The COG entries identified were primarily associated with key cellular processes, including translation, ribosomal structure, and biogenesis (COG0751), defence and restriction systems (COG0732), cell envelope biogenesis, outer membrane (COG1207), and inorganic ion transport and metabolism (ENO4G10XNNV). The KEGG orthologue primarily involved glycosaminoglycan degradation, amino sugar and nucleotide sugar metabolism (K01154), highlighting a broad range of biochemical activities that these genes influence, ranging from basic cellular structure and energy management to more specific interactions with the cellular environment and defence mechanisms (Fig. 7).

## Discussion

This study shows the complex composition and functional roles of the ewe vaginal microbiota, offering insights into its diversity, structure, and metabolic potential. Our findings highlight the intricate interactions between microbial taxa and other factors potentially influenced by the environment and the host genetics, as well as their potential influence on reproductive health. In this discussion, we address the key aspects of the microbiota composition, the functional capabilities identified, and the diversity observed across different groups.

### Vaginal microbiota composition

Our compositional results align partially with previous studies. At the phylum level, we identified Bacillota (Firmicutes), Pseudomonadota, Bacteroidota, Fusobacteriota, Actinomycetota, Mycoplasmatota, Campylobacterota, Spirochaetota, Nucleocytoviricota, and Ascomycota as the most abundant taxa, consistent with the findings of Geenwood et al. [29] and Reinoso-Peláez et al. [27], highlighting the prevalence of these taxa in the ewe’s vaginal core microbiome. At the genus level, we observed similarities in *Staphylococcus, Escherichia, Histophilus, Mycoplasma, Fusobacterium, Actinobacillus*, and *Corynebacterium*, as also reported by Reinoso-Peláez et al. [27]. Furthermore, when comparing our results with our previous study on 16S rRNA amplicon sequencing [35], we confirmed that *Histophilus, Fusobacterium, Parvimonas, Bacteroides,* and *Streptococcus* are components of the core microbiome, demonstrating the consistency of these results across different methodologies.

However, we also noted significant differences with previous studies. *Staphylococcus* was the most abundant genus in this study, and *Campylobacter*, *Anaplasma*, and *Enterococcus* were present in over 90% of our samples—an abundance not reported in the core of our previous studies [23, 27–29]. These differences may be influenced by several factors, including methodology, database, or host contamination. This study employed long-reading metagenomic sequencing with protein mapping, in contrast to our previous study and others mentioned that utilised 16S rRNA amplicon sequencing. The NCBI nr database used here is extensive but less specialised compared to the highly curated 16S rRNA databases (e.g. SILVA and Greengenes) [51, 52]. Sequencing microbial flora from vaginal tissues without prior PCR amplification posed a significant challenge due to substantial host contamination, as also reported by the Human Microbiome Project Consortium [53]. It was evident in our study that 5% of the sequenced reads were microbial, with 64% of these corresponding to unclassified genera (Fig. 2). These conditions could potentially provide a more accurate representation of microbial proportions but also dilute microbial DNA, reducing the sequencing depth and coverage.

Several of the core genera identified have been frequently reported in other ruminant body compartments. *Histophilus* and *Mycoplasma*, for example, are recognised inhabitants of the respiratory tract in ruminants [54, 55], while *Bacteroides* and *Fusobacterium* often predominate in the rumen or hindgut [56, 57]. Although direct “cross-talk” between body sites is not fully understood, these overlaps suggest that immune factors, environmental exposures, and management practices may shape microbial composition across multiple mucosal surfaces [58, 59]. Nonetheless, since our data are resolve at the genus level, it is possible that distinct species are colonising the respiratory and reproductive tracts. Future research involving strain-level analyses may better clarify the true extent of inter-site transmission and its role in ewe fertility.

### Microbial diversity across host and environmental factors

In general, alpha diversity varied significantly across herds. Latxa exhibited the lowest diversity for the Observed and Chao1 indices but showed high Shannon and InvSimpson values, suggesting that the taxa present heterogeneous abundance and evenness, increasing these indices. An interesting result observed in Latxa is that while the antibiotic treatment used may have contributed to reduce the number of taxa, the pregnancy rate of this group was the highest, which could suggest that the antibiotic is avoiding the proliferation of pathogenic taxa. However, this increase might also be influenced by the semen preparation protocols. In our previous study using a 16S rRNA amplicon approach for taxonomic identification [35], Latxa had the lowest alpha diversity in both Observed and Shannon values for amplicon sequence variant (ASV) classification, which differs from the higher Shannon index in this study. This difference may reflect the previously discussed effect of antibiotics amplifying methodological biases; the 16S rRNA metabarcoding approach, reliant on PCR amplification, tends to overrepresent dominant taxa and underdetect rare ones, resulting in lower equitability and Shannon values. In contrast, long-reading metagenomics captures all available DNA, including less abundant taxa. Regarding pregnancy, differences in alpha diversity were not significant, in line with our analogous 16S approach [35] and others like Zhang et al. [60], Koester et al. [23], and Reinoso-Peláez et al. [27]. Older ewes and those with higher parity exhibit lower alpha diversity, suggesting that ageing and repeated parturitions may impact microbial community stability, possibly due to physiological or immune changes. However, as the differences were subtle, this effect might not be strong or could be influenced by other factors such as individual variability, environment, or management practices.

The beta diversity and RF prediction analysis revealed notable differences among breeds and herds, consistent with our previous results [35]. PERMANOVA results also confirmed significant differences in the microbiota between herds and breeds, indicating that herds of the same breed tend to be more similar to each other than to those of other breeds. This pattern may reflect both genetic and environmental influences. Although research directly linking host genetics to reproductive microbiota remains limited, studies such as those by Li et al. [61] and Wang et al. [62] have identified associations between host genetics and microbiota from other sources such as rumen, in cattle and sheep. In terms of environmental influences, studies by Reinoso-Peláez et al. [27] and Koester et al. [23] have shown that factors such as PRID use and changes during gestation significantly affect the vaginal microbiota in ewes. In this study, only two herds were sampled for the same breed, so the design had limitations. However, herds of the Manchega breed differ in their management practices. The MNR herd is non-commercial and primarily dedicated to research activities, while MVL is a commercial with management practices tailored to optimise economic production. The similarity observed in the PCA between the MNR and MLV herds of Manchega could be attributed to stronger genetic links between animals of the same breed. So, a genetic component associated with the host may also be influencing the microbiota composition of the animals.

Significant differences related to pregnancy status were detected in the global model, confirming that microbiota composition may influence, to some extent, pregnancy. Random forest also showed moderate predictive accuracy for pregnancy (56.11% at the genus level), but given that this is a binary classification (pregnant vs. non-pregnant), this accuracy is only slightly above the 50% expected if predictions were random. These results suggest a differential effect of microbiota on pregnancy, but the predictive signal might reside in more specific taxonomic groups rather than broad community shifts. In contrast, age and parity—both with five categories—had lower RF accuracy (~ 40%), yet this remains considerably higher than the 20% expected by chance, indicating the model captures meaningful variation. When treated as continuous variables, age showed a stronger RF correlation than parity (*r* = 0.47 vs. *r* = 0.34 at the genus level), and the model achieved a RMSE of approximately 2 years, suggesting a moderate ability to distinguish younger from older animals based on microbial composition. This aligns with PERMANOVA results, where older animals (more than 5 years and more than 3 parturitions) exhibited distinct microbiota composition, suggesting that cumulative physiological changes associated with ageing and reproductive history may drive microbial shifts. However, given the high correlation between age and parity (*r* = 0.94), it remains challenging to disentangle their individual contributions. Moreover, parity is more susceptible to errors in farm records, particularly when integrating new animals with incomplete reproductive histories.

Although the PCA pattern did not solve the separation of functional pathways between groups, PERMANOVA identified significant differences between groups in COG and KEGG categories. This functional redundancy makes it challenging for PCA to separate microbiome samples based solely on COG or KEGG functions, as similar functions can be fulfilled by different community compositions.

### Differential abundance analysis

*Treponema, Caviibacter, Peptoniphilus, Helcococcus, Actinomyces, Gemella, Trueperella*, and *Phocaeicola*, more abundant in non-pregnant ewes, have limited records in the ewe reproductive microbiota literature. However, species such as *Treponema pallidum* is the causative agent of significant human diseases like syphilis [63]. *Caviibacter abscessus* is associated with purulent vaginal discharge in cattle [21] and cervical abscesses in guinea pigs [64]. *Peptoniphilus* has been found in higher abundance in individuals failing to establish pregnancy [65]. *Helcococcus* has been identified as a potential indicator of metritis [66]. *Actinomyces* species have been linked to reproductive tract infections in cattle [67], and their abundance appears to vary with age in rams [28]. *Gemella asaccharolytica* has been linked to increased odds of HIV acquisition in humans [68]. *Trueperella pyogenes* have been recognised as opportunistic pathogens causing metritis and mastitis in other livestock species [69]. Finally, an increase in *Phocaeicola* abundance has been reported in cases of metritis in dairy cows [70], suggesting its potential involvement in uterine infections.

The higher abundance of these genera in non-pregnant ewes could reflect a dysbiotic state or a less conducive environmental conditions to pregnancy success, potentially contributing to adverse reproductive outcomes.

The high prevalence of the genus *Streptobacillus* (exceeding 80%, Supplementary material 2: Fig. S8), although not part of the core microbiota, suggests that it may play more transient or condition-specific roles within the vaginal microbiome. This genus was also identified by Swart [71] as one of the most abundant in the vaginal microbiota of ewes, suggesting that it is a stable and common component of these microbial communities. Despite its abundance, there is no previous record explicitly linking *Streptobacillus* to negative reproductive outcomes in ewes. However, specific species within this genus may have a negative impact on pregnancy; *Streptobacillus notomytis* was 19 times more abundant (*p-*adjusted = 0.05) in non-pregnant than in pregnant ewes in a previous study by our group [28].

In our study, we found some genes significantly more abundant in non-pregnant ewes (Fig. 5), that were mainly identified in *Streptobacillus* (Fig. 6), that play crucial roles in defence systems. For example, *COG0732*, associated with the restriction endonuclease S subunit, suggests a defensive microbial community potentially creating a hostile environment less conducive to pregnancy. This aligns with findings by Wang et al. [34] and Merk [72], which found this COG and highlight the significance of defence genes in microbial adaptation and competition in human and multiple procaryotic species, respectively. The gene *K01154*, which encodes the Type I restriction enzyme S subunit (Fig. 7), is notable for its role in bacterial defence mechanisms, particularly in restricting foreign DNA. This observation aligns with studies by Freese et al. [73] on microbial defence systems founded in *Phaeobacter gallaeciensis* from the Pacific Ocean, suggesting that the presence of *K01154* might contribute to a more antagonistic environment, potentially impacting fertility. Finally, the genera *Histophilus*, *Mycoplasmopsis*, *Fusobacterium*, *Bacteroides*, and *Campylobacter* were significantly more abundant in non-pregnant ewes across different herds and were also identified within the core microbiota (Figs. 2 and 5; Supplementary material 2: Figs. S8–S12). These genera were similarly reported as more abundant in non-pregnant ewes by Kirkbride [74], Serrano et al. [28], Koester et al. [23], Yaeger et al. [75], and Reinoso-Peláez et al. [27]. These findings suggest that in non-pregnant ewes, an overabundance of certain taxa from the core microbiota may lead to a dysbiosis state, contributing to reproductive challenges. Although these genera could contain both beneficial and pathogenic species, their increased abundance above normal levels could cause harmful effects. Environmental and genetic factors, such as hormonal changes and immune activity, likely play a role in this imbalance, disrupting the microbial environment necessary for successful pregnancy [21, 23, 27, 28, 76]. Key species like *Histophilus somni, Campylobacter fetus, Campylobacter jejuni, and Campylobacter coli* have been associated with adverse reproductive outcomes such as abortion, vaginitis, polyarthritis/tenosynovitis, and epididymitis in sheep [28, 75, 77, 78]. Similarly, some *Bacteroides* species, typically commensal, can become opportunistic pathogens and have been linked to gynaecological infections in humans [79]. Species like *Bacteroides heparinolyticus* and *Bacteroides pyogenes*, along with *Fusobacterium necrophorum*, have been associated with metritis and clinical endometritis in dairy cows [80–83], indicating their potential role in reproductive disorders. *Mycoplasmopsis *species have been detected in both healthy and infertile cattle, with a higher prevalence in animals with reproductive issues [84]. This suggests that certain *Mycoplasmopsis *taxa could act as opportunistic pathogens, contributing to reproductive dysfunction under specific conditions.

These genera were also associated with several genes that were significantly more abundant in non-pregnant ewes in our study. For instance, *ENOG410XNNV*, mainly expressed by *Bacteroides* (Bacteroidota), is involved in ion transport, supporting the idea that these bacteria help maintain ion balance, which could influence the environment needed for pregnancy. This aligns with Qi et al.[85], who identified this gene and pathway in the cecal microbiota of broilers and layers. COG1207, mainly found in *Histophilus* and *Fusobacterium*, suggests these bacteria strengthen their cell walls, aiding their survival in the vaginal environment. COG0751, linked to *Fusobacterium*, is crucial for protein synthesis, suggesting a higher need for efficient protein production in non-pregnant-related bacteria, as highlighted by Pragya et al. [86] in their studies with *Lactobacillus fermentum*.

There were both agreements and discrepancies between this study and our analogous study using the 16S rRNA amplicon approach, likely due to their distinct methodologies. 16S rRNA sequencing targets the conserved 16S rRNA gene, a taxonomic marker for Bacteria and Archaea identification which has a lower power for species identification. In contrast, ONT metagenomic sequencing offers a more comprehensive view by detecting a wider range of organisms, including Fungi and viruses. Its longer reads also allow for functional profiling through gene and metabolic pathway analysis (COG and KEGG), providing insights into the community’s functional potential. Despite these advantages, ONT has a higher sequencing error rate and is more prone to host DNA contamination, particularly in samples such as those in this study, where removing large amounts of host DNA is difficult. This, combined with the complexity of vaginal microbial communities —often underrepresented in databases— posed significant challenges for species-level classification. As a result, we focused on genus-level assignments and their functional genes. Consequently, strict filtering protocols and advanced bioinformatics approaches are needed for accurate taxonomic and functional classification. This complexity can complicate microbial classification and contribute to taxonomic discrepancies when using different databases. Despite these differences, our findings underscored the robustness of our approach as both beta diversity and differential abundance analyses consistently identified similar taxa associated with pregnancy, thereby further validating our previous results across different sequencing technologies. Combining both methodologies enhances our understanding of microbial communities, leveraging the high precision of 16S rRNA and the broader coverage of ONT.

## Conclusion

In this study, the microbial diversity of the ewe’s vaginal microbiome was significantly influenced by both herd and breed factors. Despite our experimental design limiting the ability to accurately distinguish the relative contributions of genetic factors associated with breed and environmental factors linked to management practices, it seems that both influence the microbial diversity and abundance. While the overall microbiota showed a subtle effect on pregnancy rate, specific genera were significantly more abundant in non-pregnant ewes. These included *Histophilus, Mycoplasmopsis, Fusobacterium, Bacteroides, Campylobacter, Streptobacillus, Trueperella, Gemella, Peptoniphilus, Helococcus, Actinomyces, Caviibacter, Treponema*, *Tissierella*, and *Phocaeicola* suggesting that these particular taxa may harbour pathogenic traits or elicit inflammatory responses that could contribute to unsuccessful pregnancy outcomes. This finding highlights the importance of microbial composition in reproductive health, as certain bacteria may disrupt the uterine environment or negatively interact with the host’s immune system, ultimately impairing fertility and pregnancy maintenance. Several of these taxa belonged to the core microbiota, and an imbalance in their typical abundance may be associated with creating a less favorable environment for successful reproduction. Age and parity were also associated with microbiota composition, particularly in ewes older than five years or with more than three parturitions. The ability of RF models to moderately predict age suggests that cumulative physiological changes may contribute to microbial shifts over time.

The long-reading metagenomic approach implemented here enabled not only taxonomic classification, but also detailed functional analysis based on gene families, metabolic pathways, and biochemical activities. Functional pathways identified here as more abundant in non-pregnant ewes were primarily linked to carbohydrate metabolism, defence mechanisms, and structural resilience, raising the possibility that these microbial functions may create a less hospitable environment for embryo implantation and development. This approach provides deeper insights into the microbiome's role in reproductive outcomes.

## Supplementary Information

Supplementary material 1: Automated Workflow for SqueezeMeta Analysis at CESGA

Supplementary material 2: Figure S1. Alpha diversity measures (Observed, Chao1, Shannon, and InvSimpson index) for Pregnancy and herd at the Genus (a) and Phylum (b) level. Figure S2. Alpha diversity measures (Observed, Chao1, Shannon, and InvSimpson index) for Pregnancy at the Genus (a) and Phylum (b) level. Figure S3. Alpha diversity measures (Observed, Chao1, Shannon, and InvSimpson index) for age category at the genus (a) and phylum (b) level. Figure S4. Alpha diversity measures (Observed, Chao1, Shannon, and InvSimpson index) for parity category at the genus (a) and phylum (b) level. Figure S5. Rarefaction curves for all analysed samples for alpha diversity analysis. Figure S6. Principal component analysis of microbiota composition by Age (a, b) and Parity (c, d) categories. Figure S7. Heatmap representing the RA of taxa against group means (on a 100-point scale) from the differential abundance analysis for pregnancy, for the global model (ALL) and within each herd. Figure S8. Prevalence of pregnancy by herd groups. Figure S9. Boxplots of taxa with significant differential abundance between pregnant and non-pregnant ewes for Histophilus, Mycoplasmopsis, Campylobacter, and Bacteroides genera. Figure S10. Boxplots of taxa with significant differential abundance between pregnant and non-pregnant ewes for Fusobacterium, Streptobacillus, Gemella, and Trueperella genera. Figure S11. Boxplots of taxa with significant differential abundance between pregnant and non-pregnant ewes for Peptoniphilus, Caviibacter, Actinomyces, and Helococcus genera. Figure S12. Boxplots of taxa with significant differential abundance between pregnant and non-pregnant ewes for Treponema, Tissierella, and Phocaeicola genera. Figure S13. Boxplots of taxa with significant differential abundance between pregnant and non-pregnant ewes for Fusobacteriota, Mycoplasmatota, Campylobacterota, and Uroviricota phyla. Figure S14. Boxplots of taxa with significant differential abundance between pregnant and non-pregnant ewes for COG0732, COG0751, COG1207, and ENOG410XNNV. Pregnancy-positive samples in blue and pregnancy-negative samples in red. Figure S15. Boxplots of taxa with significant differential abundance between pregnant and non-pregnant ewes for KO1154. Table S1. PERMANOVA results for the effects of Pregnancy, age category, and parity category on microbiota composition within each herd group at genus level. Table S2. Pairwise comparisons for Herd groups using permutation MANOVAs on a distance matrix. Table S3. Pairwise comparisons for Age category groups using permutation MANOVAs on a distance matrix. Table S4. Pairwise comparisons for Parity category groups using permutation MANOVAs on a distance matrix

## Data Availability

Sequence data that support the findings of this study have been deposited in the NCBI Sequence Read Archive (SRA) under the BioProject accession number PRJNA1207962. These data can be accessed at the following link: https://www.ncbi.nlm.nih.gov/sra/PRJNA1207962.
