# Supplementary material for: The influence of vaginal microbiota on ewe fertility: a metagenomic and functional genomic approach
Source: Microbiome. 2025 Aug 1;13:177. doi: 10.1186/s40168-025-02165-z (PMC12315406; doi:10.1186/s40168-025-02165-z)
Supplement: Supplementary file 2 — Supplementary material 1: Automated Workflow for SqueezeMeta Analysis at CESGA [file 40168_2025_2165_MOESM1_ESM.pdf]

Automated Workflow for SqueezeMeta Analysis at CESGA

This document provides a detailed description of the automated workflow implemented to process metagenomic data using SqueezeMeta at CESGA (Centro de Supercomputación de Galicia). Due to the high computational demand of analysing 297 samples of vaginal exudate, we developed a custom workflow using Bash and Snakemake for job management and submission within the SLURM scheduling system.

The following sections describe the scripts used in this workflow, their purpose, and their structure, ensuring that the entire bioinformatics pipeline is documented and reproducible.

1. **Main Workflow Script: run_SqueezeMeta_pipeline.sh**

This script is responsible for initiating the SqueezeMeta workflow. It loads the necessary computational environment, checks for previous runs, and submits jobs dynamically to SLURM, managing queue limits and monitoring job completion.

Script: `run_SqueezeMeta_pipeline.sh`

```
#!/bin/bash

# Load the required environment
module load cesga/system miniconda3/22.11.1-1
conda activate SqueezeMeta

# Create a directory for logs if it does not exist
mkdir -p ./slurm_and_maker_log

# Ensure the Snakefile_SqueezeMeta has been executed before
proceeding
if [ ! "$(ls -A $SQM_SCRIPTS_DIR)" ]; then
    num_logs=$(ls ./slurm_and_maker_log/Snakefile_SqueezeMeta_*.log
2> /dev/null | wc -l)
    module load snakemake
    snakemake -s ./Snakefile_SqueezeMeta --cores 1 >
./slurm_and_maker_log/Snakefile_SqueezeMeta_$((num_logs+1)).log 2>&1
fi

# Define the folder containing the SqueezeMeta scripts
SQM_SCRIPTS_DIR="sbatch/SqueezeMeta/"

# Get the list of scripts to be executed
sqm_scripts=$(ls $SQM_SCRIPTS_DIR | sed 's/.sh//g')

# Check the queue and completed jobs
queue_jobs=$(squeue -o "%j" --noheader)
completed_jobs=$(ls ../results/sqm_longreads/ | sed 's/sqm_//g')

# Convert names to arrays
sqm_scripts=($sqm_scripts)
queue_jobs=($queue_jobs)
completed_jobs=($completed_jobs)

# Set job submission limit
current_jobs=$(squeue --noheader | wc -l)
let "available_slots = 50 - current_jobs"
```

```
if [ "$available_slots" -le 0 ]; then
    echo "The queue is full."
else
    for file in "${sqm_scripts[@]}"; do
        if [[ ! " ${completed_jobs[@]} " =~ " ${file} " ]] && [[ ! " ${queue_jobs[@]} " =~ " ${file} " ]] && (( to_submit_count < available_slots )); then
            sbatch $SQM_SCRIPTS_DIR$file.sh
            ((to_submit_count++))
        fi
    done

    if (( to_submit_count == 0 )); then
        echo "All jobs have either been submitted or are already completed."
    else
        echo "$to_submit_count jobs have been submitted. Some jobs might still be pending for next submission."
    fi
fi
```

2. Snakemake Workflow: Snakefile_SqueezeMeta

This Snakemake script automates the creation of batch job scripts for each sample, ensuring that:

1. Directories and input files are correctly structured.
2. SLURM batch scripts are automatically generated for each sample.
3. The entire workflow runs efficiently without manual intervention.

Script: `Snakefile_SqueezeMeta`

```
from glob import glob
import os

# Define project directories
project_dir = os.path.abspath("..")
filtered_data_dir = os.path.join(project_dir, "results", "filtered_data")
SAMPLES = [os.path.basename(s).replace('.fastq', '') for s in glob(os.path.join(filtered_data_dir, "*.fastq"))]

# Main workflow rule
rule all:
    input:
        expand("sbatch/SqueezeMeta/{sample}.sh", sample=SAMPLES),
        expand("../results/sqm_longreads/mydata/{sample}.txt", sample=SAMPLES)

# Rule to create required directories
rule create_dirs:
    output:
        dirs_exist = touch("../results/sqm_longreads/mydata/.dir_exists")
    shell:
        """
```

```
        mkdir -p ../results/sqm_longreads/mydata
        mkdir -p sbatch/SqueezeMeta
        """

# Rule to generate sample metadata files
rule create_sqm_files:
    input:
        create_dirs = "../results/sqm_longreads/mydata/.dir_exists",
        sample_file = "../results/filtered_data/{sample}.fastq"
    output:
        "../results/sqm_longreads/mydata/{sample}.txt"
    shell:
        """
        sample_name=$(basename {input.sample_file})
        echo -e "{wildcards.sample}\t$sample_name\tpair1" > {output}
        """

# Rule to create SLURM batch job scripts
rule create_sbatch_scripts:
    input:
        sample_file = "../results/filtered_data/{sample}.fastq"
    output:
        "sbatch/SqueezeMeta/{sample}.sh"
    shell:
        """
        echo "#!/bin/bash" > {output}
        echo "#SBATCH -N 1" >> {output}
        echo "#SBATCH -n 1" >> {output}
        echo "#SBATCH -c 12" >> {output}
        echo "#SBATCH -p thinnodes" >> {output}
        echo "#SBATCH -t 24:10:00" >> {output}
        echo "#SBATCH -J {wildcards.sample}" >> {output}
        echo "#SBATCH --mem=64GB" >> {output}
        echo "#SBATCH --mail-type=END" >> {output}
        echo "#SBATCH --mail-user=user@example.com" >> {output}
        echo "#SBATCH -o
slurm_and_maker_log/host_filter_{wildcards.sample}.out" >> {output}
        echo "" >> {output}
        echo "module load miniconda3" >> {output}
        echo "conda activate SqueezeMeta" >> {output}
        echo "sqm_longreads.pl -p
../results/sqm_longreads/sqm_{wildcards.sample} -s
../results/sqm_longreads/mydata/{wildcards.sample}.txt -f
../results/filtered_data --euk -t 12" >> {output}
        """
```

SLURM Job Submission Example

Each sample is processed using a dedicated SLURM job script. The following is an example of how a sample is submitted for processing.

```
Example SLURM Job Script:

#!/bin/bash
#SBATCH -N 1                # Number of nodes
#SBATCH -n 1                # Number of tasks
#SBATCH -c 12               # Number of CPU cores per task
#SBATCH -p thinnodes        # Partition to submit the job
```

```
#SBATCH -t 24:10:00         # Time limit (hh:mm:ss)
#SBATCH -J SAMPLE_NAME      # Job name (to be replaced by actual
sample name)
#SBATCH --mem=64GB          # Memory allocation
#SBATCH --mail-type=END     # Notification settings
#SBATCH --mail-user=user@example.com  # Email for job notifications
#SBATCH -o slurm_and_maker_log/sample_output.log  # Output log file

# Load environment and activate SqueezeMeta
module load miniconda3
conda activate SqueezeMeta

# Execute SqueezeMeta long-read analysis with eukaryotic detection
enabled
sqm_longreads.pl -p ../results/sqm_longreads/sqm_SAMPLE_NAME -s
../results/sqm_longreads/mydata/SAMPLE_NAME.txt -f
../results/filtered_data --euk -t 12
```

Finally, when the processing of all samples was completed, we merged the results from all samples using the following Bash command:

```
combine-sqm-tables.py -f samples.txt -o PROBIOTICOjoin –sqmreads
```

where `samples.txt` is a text file containing the names of the result directories generated by the SLURM job scripts.
