# Supplementary material for: The influence of vaginal microbiota on ewe fertility: a metagenomic and functional genomic approach
Source: Microbiome. 2025 Aug 1;13:177. doi: 10.1186/s40168-025-02165-z (PMC12315406; doi:10.1186/s40168-025-02165-z)
Supplement: Supplementary file 3 — Supplementary material 2: Figure S1. Alpha diversity measures (Observed, Chao1, Shannon, and InvSimpson index) for Pregnancy and herd at the Genus (a) and Phylum (b) level. Figure S2. Alpha diversity measures (Observed, Chao1, Shannon, and InvSimpson index) for Pregnancy at the Genus (a) and Phylum (b) level. Figure S3. Alpha diversity measures (Observed, Chao1, Shannon, and InvSimpson index) for age category at the genus (a) and phylum (b) level. Figure S4. Alpha diversity measures (Observed, Chao1, Shannon, and InvSimpson index) for parity category at the genus (a) and phylum (b) level. Figure S5. Rarefaction curves for all analysed samples for alpha diversity analysis. Figure S6. Principal component analysis of microbiota composition by Age (a, b) and Parity (c, d) categories. Figure S7. Heatmap representing the RA of taxa against group means (on a 100-point scale) from the differential abundance analysis for pregnancy, for the global model (ALL) and within each herd. Figure S8. Prevalence of pregnancy by herd groups. Figure S9. Boxplots of taxa with significant differential abundance between pregnant and non-pregnant ewes for Histophilus, Mycoplasmopsis, Campylobacter, and Bacteroides genera. Figure S10. Boxplots of taxa with significant differential abundance between pregnant and non-pregnant ewes for Fusobacterium, Streptobacillus, Gemella, and Trueperella genera. Figure S11. Boxplots of taxa with significant differential abundance between pregnant and non-pregnant ewes for Peptoniphilus, Caviibacter, Actinomyces, and Helococcus genera. Figure S12. Boxplots of taxa with significant differential abundance between pregnant and non-pregnant ewes for Treponema, Tissierella, and Phocaeicola genera. Figure S13. Boxplots of taxa with significant differential abundance between pregnant and non-pregnant ewes for Fusobacteriota, Mycoplasmatota, Campylobacterota, and Uroviricota phyla. Figure S14. Boxplots of taxa with significant differential abundance between pregnant [file 40168_2025_2165_MOESM2_ESM.pdf]

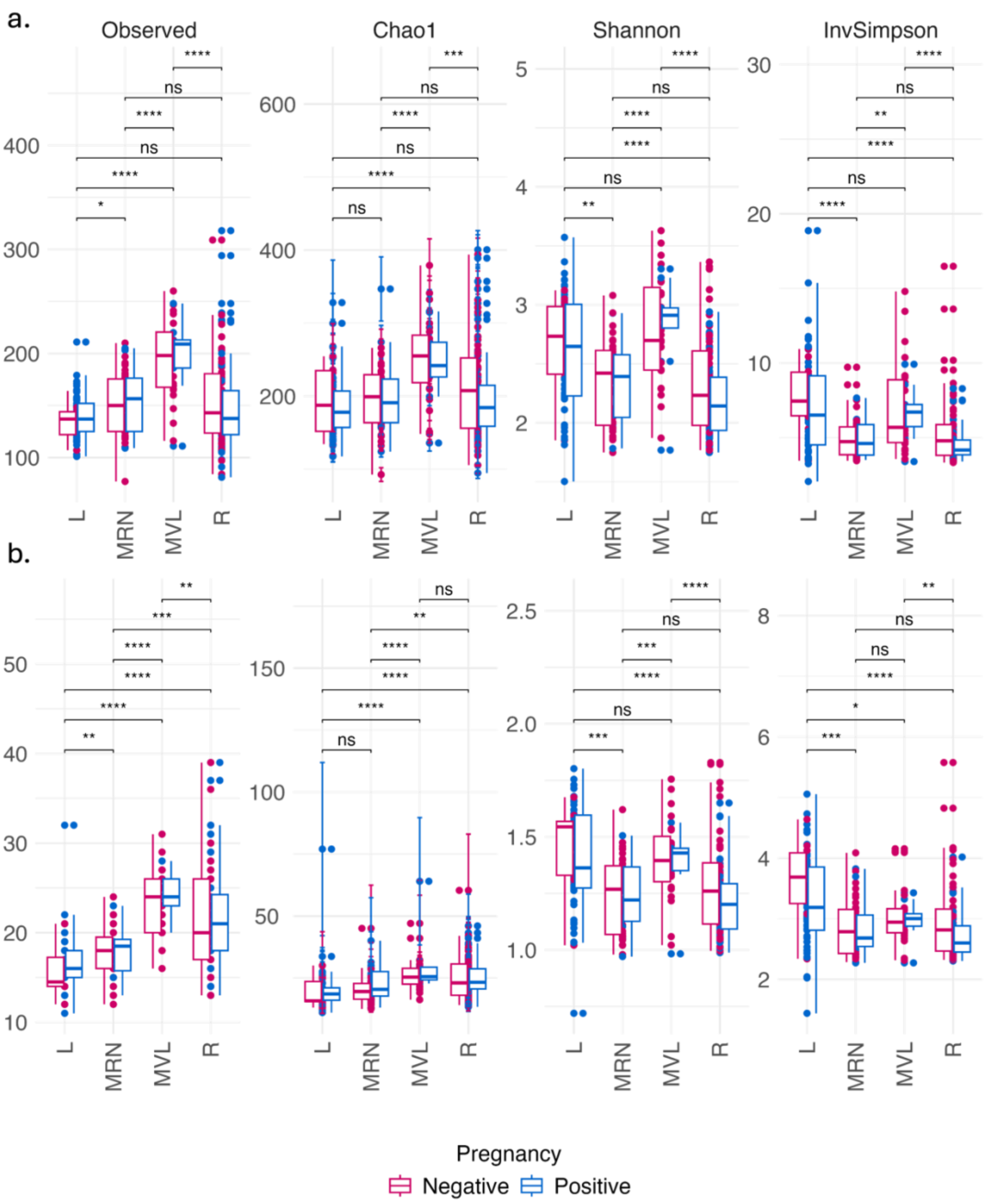

Figure S1. **Alpha diversity measures (Observed, Chao1, Shannon, and InvSimpson index) for Pregnancy and herd at the Genus (a) and Phylum (b) level**. Data were rarefied. Statistical significance is indicated by asterisks for herd comparisons: * ($p < 0.05$), ** ($p < 0.01$), *** ($p < 0.001$), **** ($p < 0.0001$). ns: not significant.

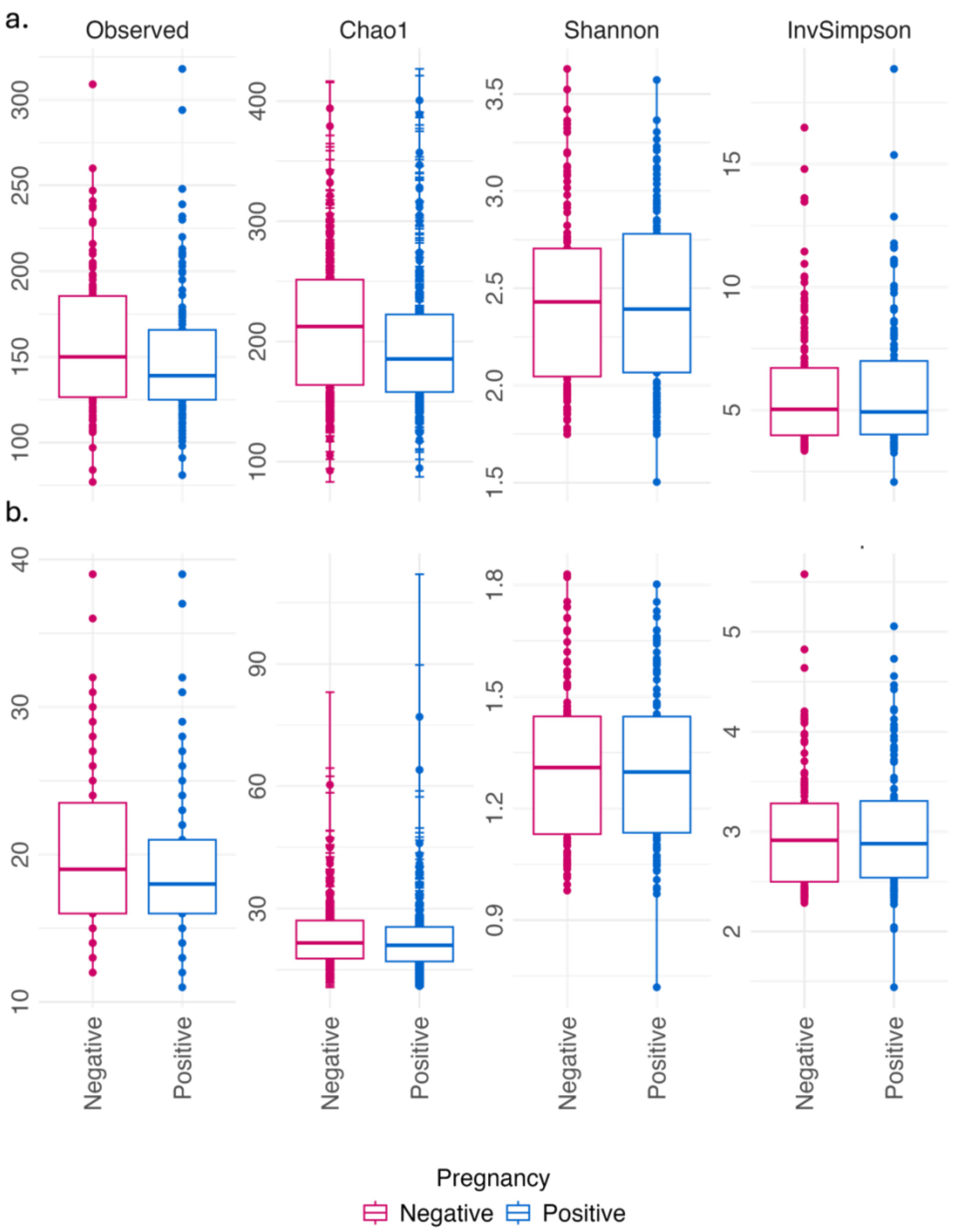

Figure S2. **Alpha diversity measures (Observed, Chao1, Shannon, and InvSimpson index) for Pregnancy at the Genus (a) and Phylum (b) level**. Data were rarefied. No significant differences were found.

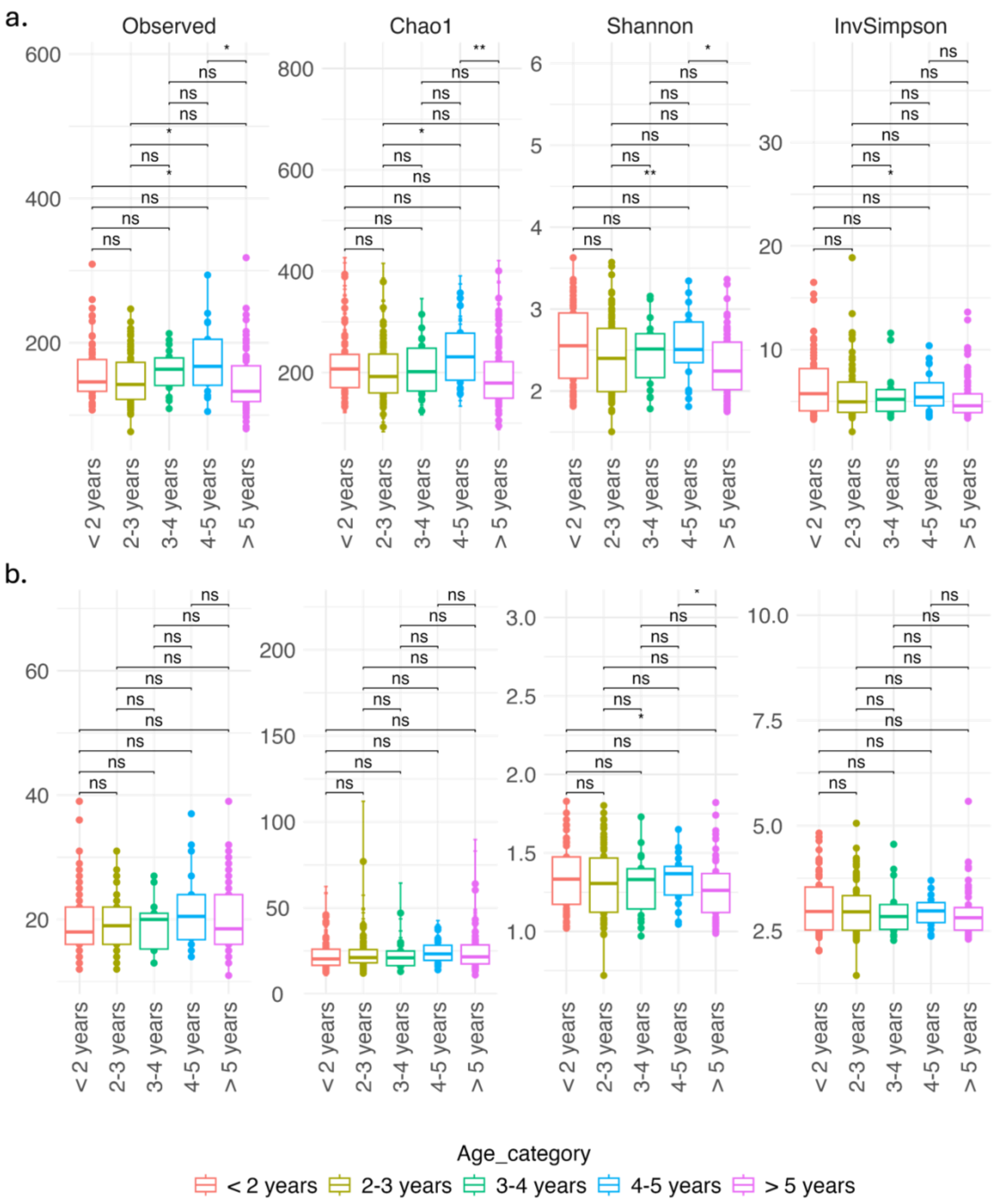

Figure S3. **Alpha diversity measures (Observed, Chao1, Shannon, and InvSimpson index) for age category at the genus (a) and phylum (b) level**. Data were rarefied. Statistical significance is indicated by asterisks: * ($p < 0.05$), ** ($p < 0.01$), *** ($p < 0.001$), **** ($p < 0.0001$). ns: not significant.

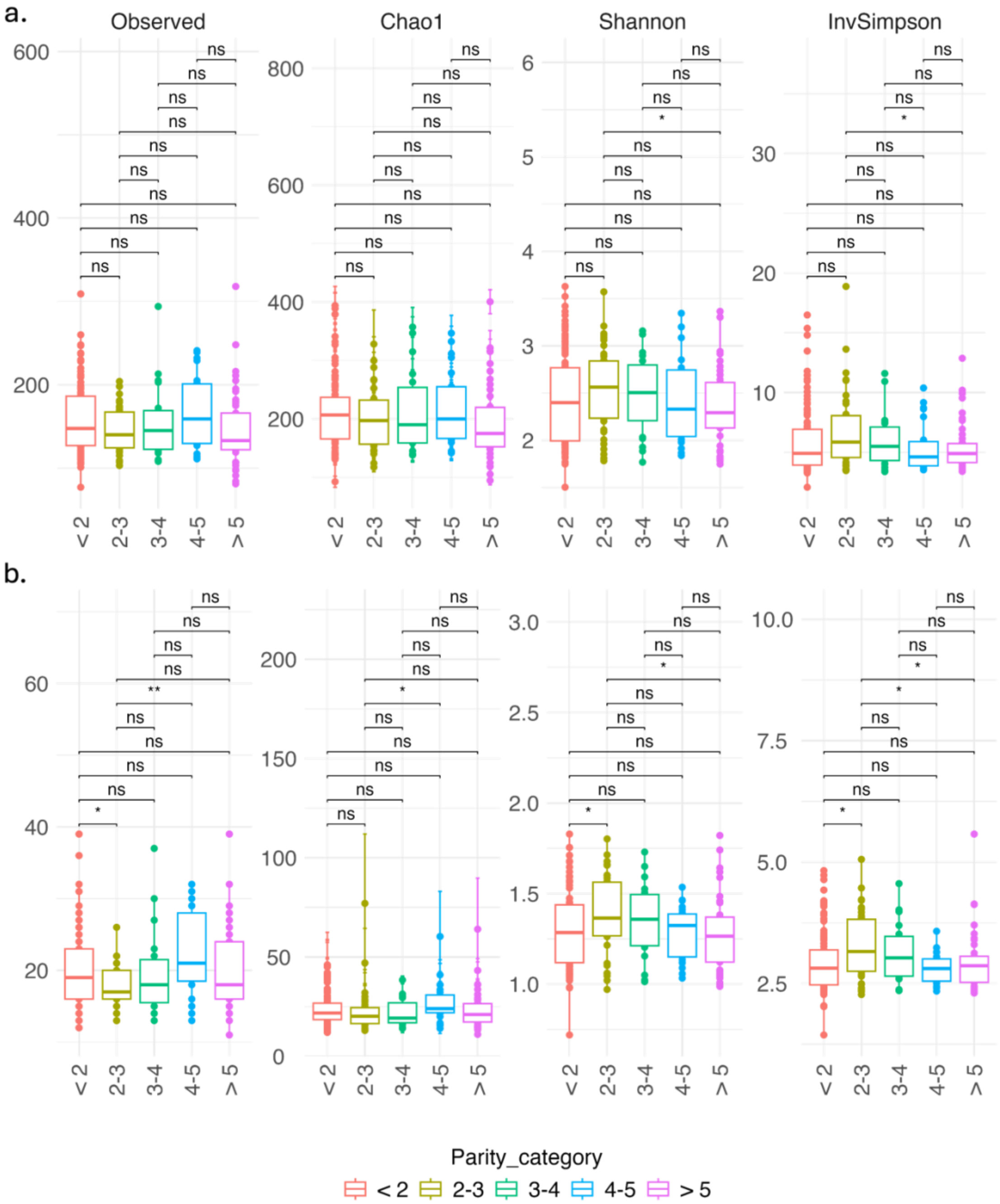

Figure S4. **Alpha diversity measures (Observed, Chao1, Shannon, and InvSimpson index) for parity category at the genus (a) and phylum (b) level**. Data were rarefied. Statistical significance is indicated by asterisks: * ($p < 0.05$), ** ($p < 0.01$), *** ($p < 0.001$), **** ($p < 0.0001$). ns: not significant.

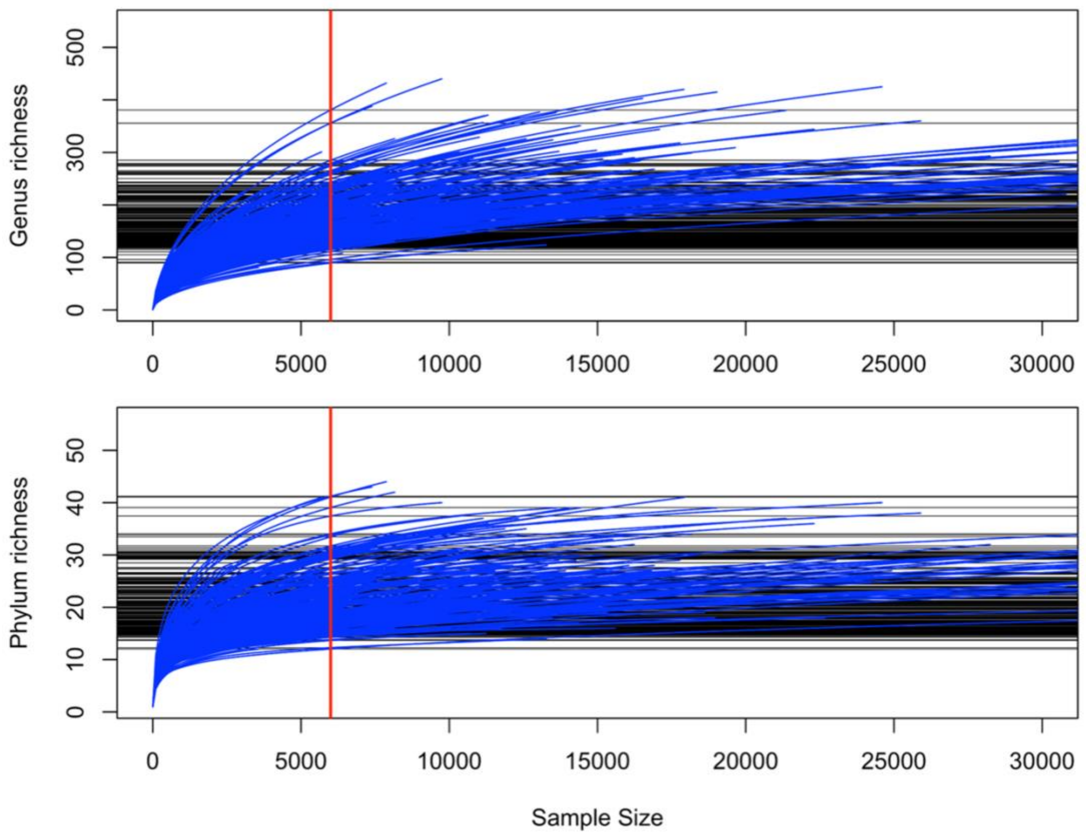

Figure S5. Rarefaction curves for all analysed samples for alpha diversity analysis. Sequencing depth in the x-axis and observed taxa richness in the y-axis for Genus and Phylum level. Blue lines represent individual samples. Red vertical dashed line indicates the threshold value (6000). black horizontal lines indicate the total sequencing depth for each sample. Black horizontal lines indicate the total sequencing depth for each sample.

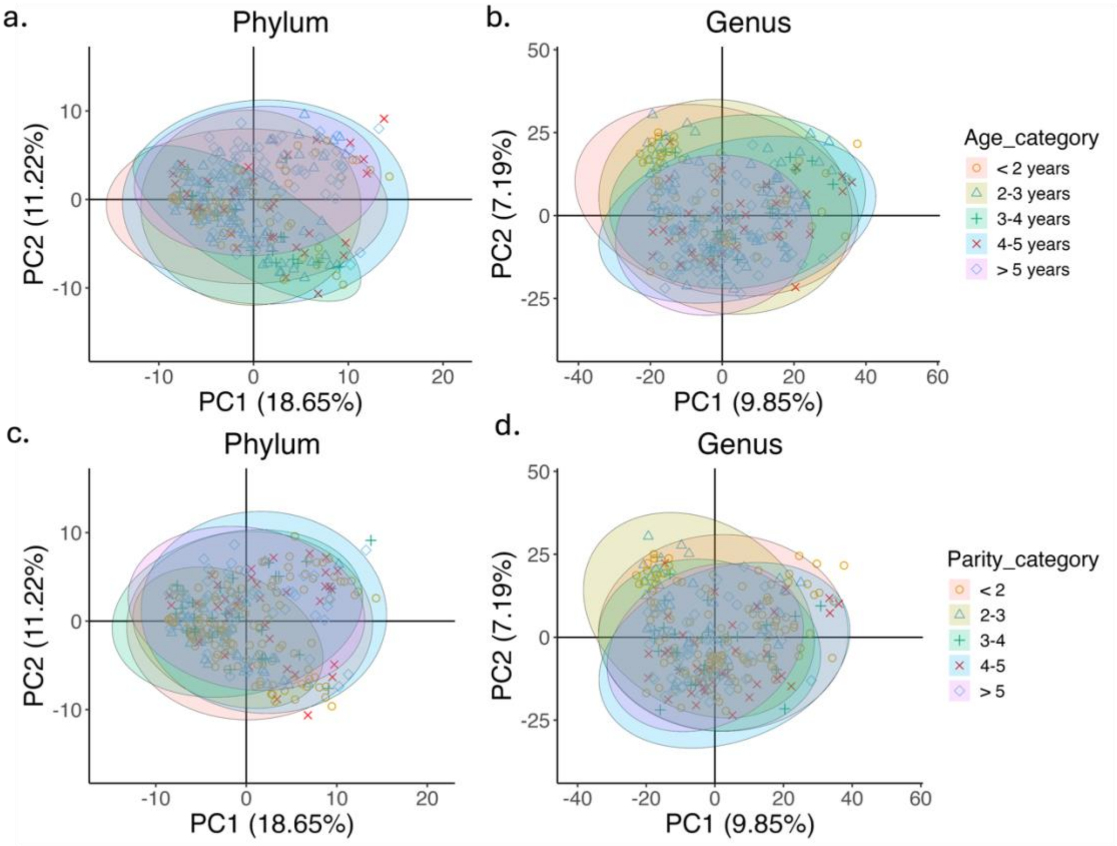

Figure S6. Principal component analysis of microbiota composition by Age (a, b) and Parity (c, d) categories. Phylum (a, c) and genus (b, d) levels were analysed. Ellipses were calculated using covariance to represent data variability within each age/parity category.

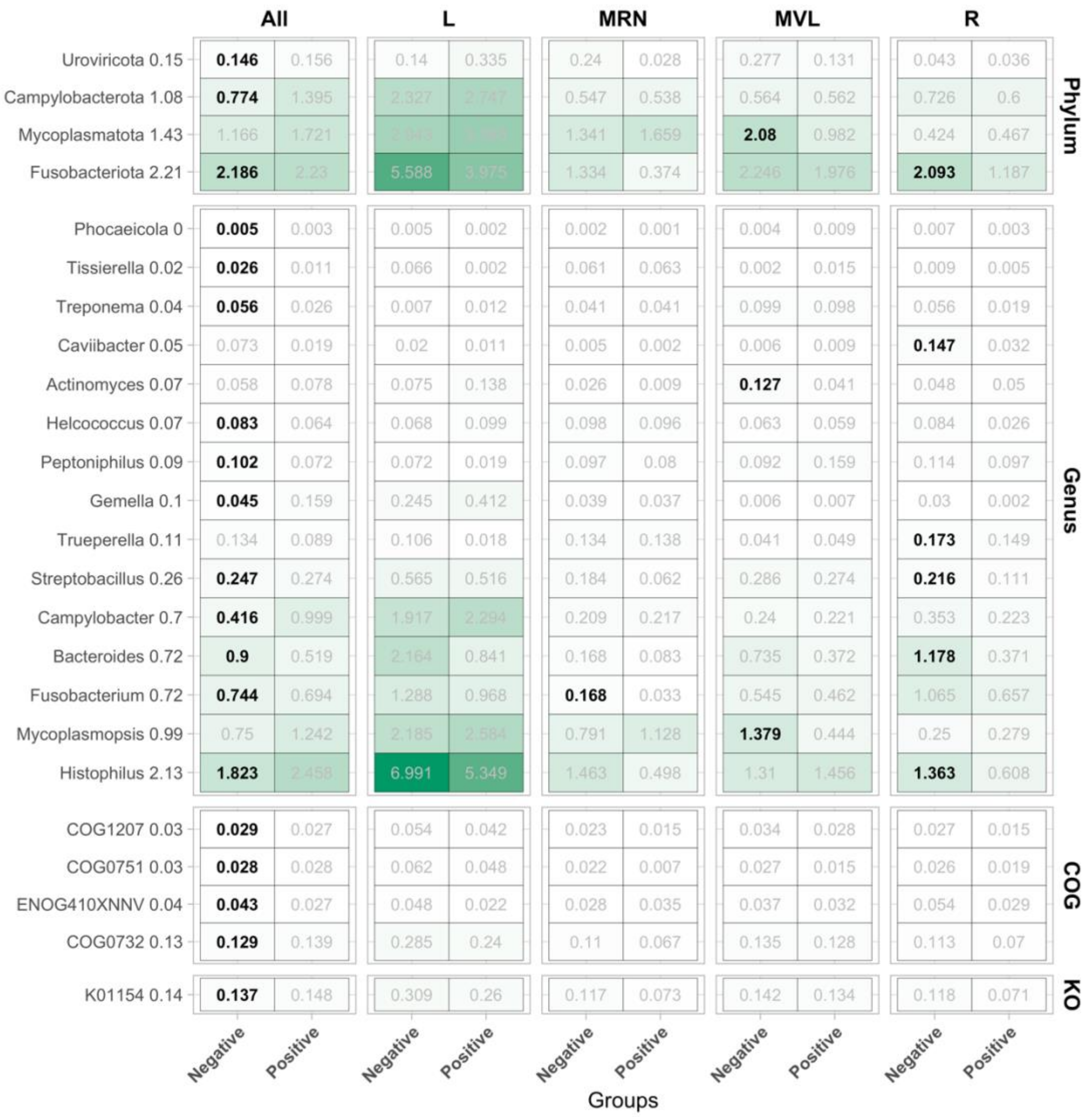

Figure S7. Heatmap representing the RA of taxa against group means (on a 100-point scale) from the differential abundance analysis for pregnancy, for the global model (ALL) and within each herd. Taxa levels include Genus, Phylum, COG, and KO. In bold, significantly differential abundant taxa between pregnant (positive) and non-pregnant (negative) ewes.

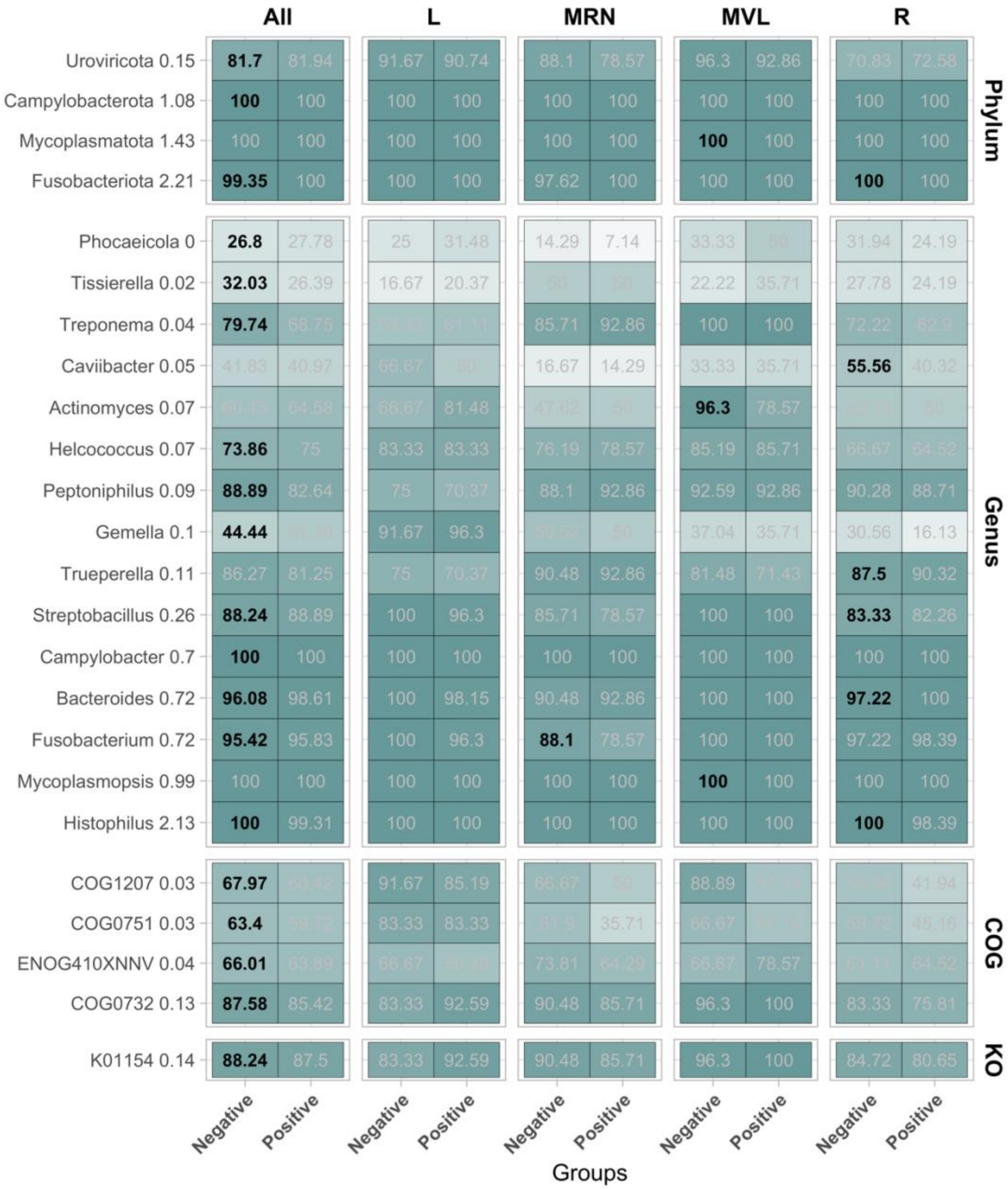

Figure S8. Prevalence of pregnancy by herd groups. Heatmap representation of prevalence of taxa normalized by group on a 100-point scale. Taxa levels include Genus, Phylum, COG and KO with significantly abundant pregnancy groups in differential analysis denoted in bold.

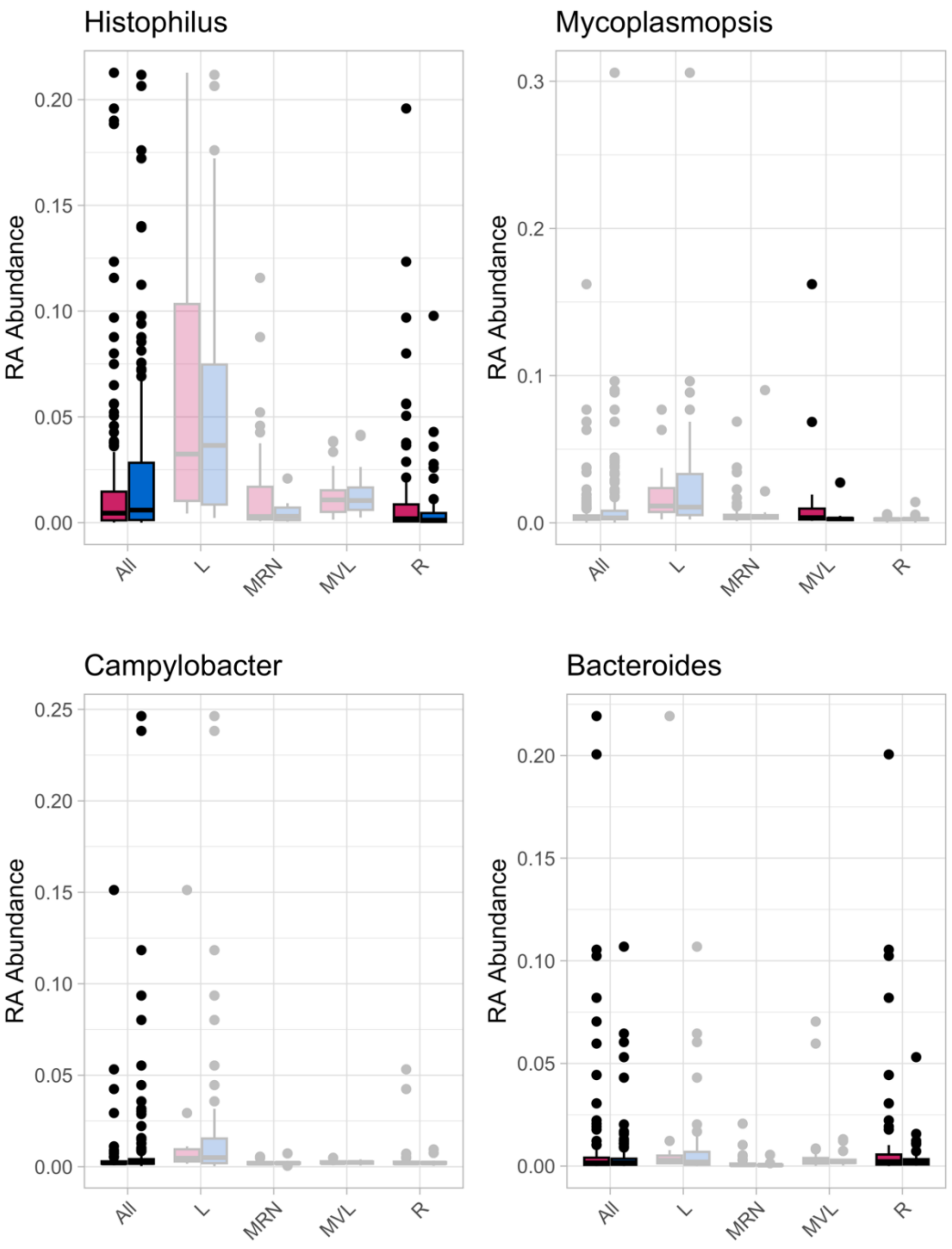

Figure S9. Boxplots of taxa with significant differential abundance between pregnant and non-pregnant ewes for *Histophilus*, *Mycoplasmopsis*, *Campylobacter*, and *Bacteroides* genera. Pregnancy-positive samples in blue and pregnancy-negative samples in red. The x-axis categorizes samples by herd level. Saturated colors represent groups with statistically significant differences; desaturated colors are used for groups without significant differences.

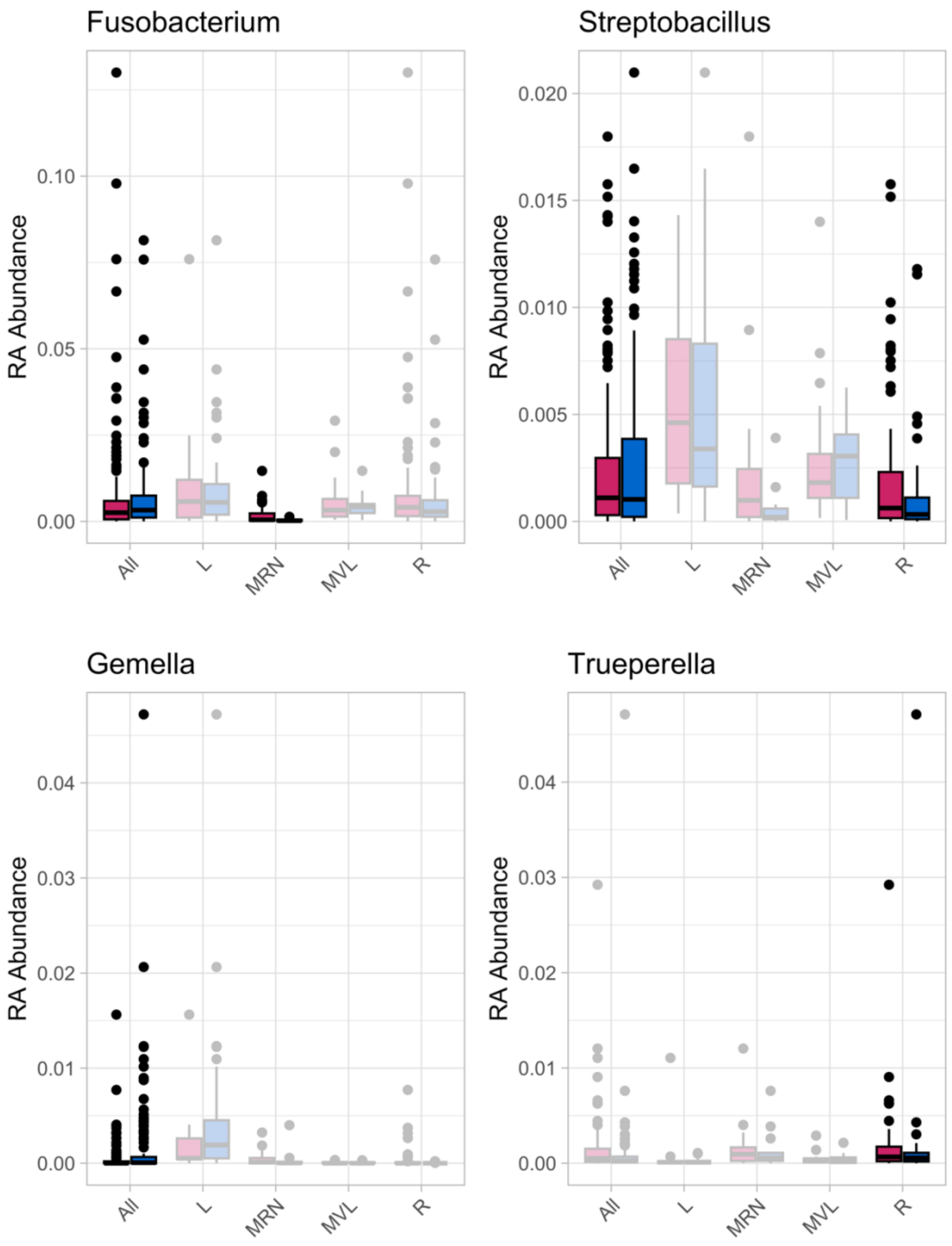

Figure S10. Boxplots of taxa with significant differential abundance between pregnant and non-pregnant ewes for *Fusobacterium*, *Streptobacillus*, *Gemella*, and *Trueperella* genera. Pregnancy-positive samples in blue and pregnancy-negative samples in red. The x-axis categorizes samples by herd level. Saturated colors represent groups with statistically significant differences; desaturated colors are used for groups without significant differences.

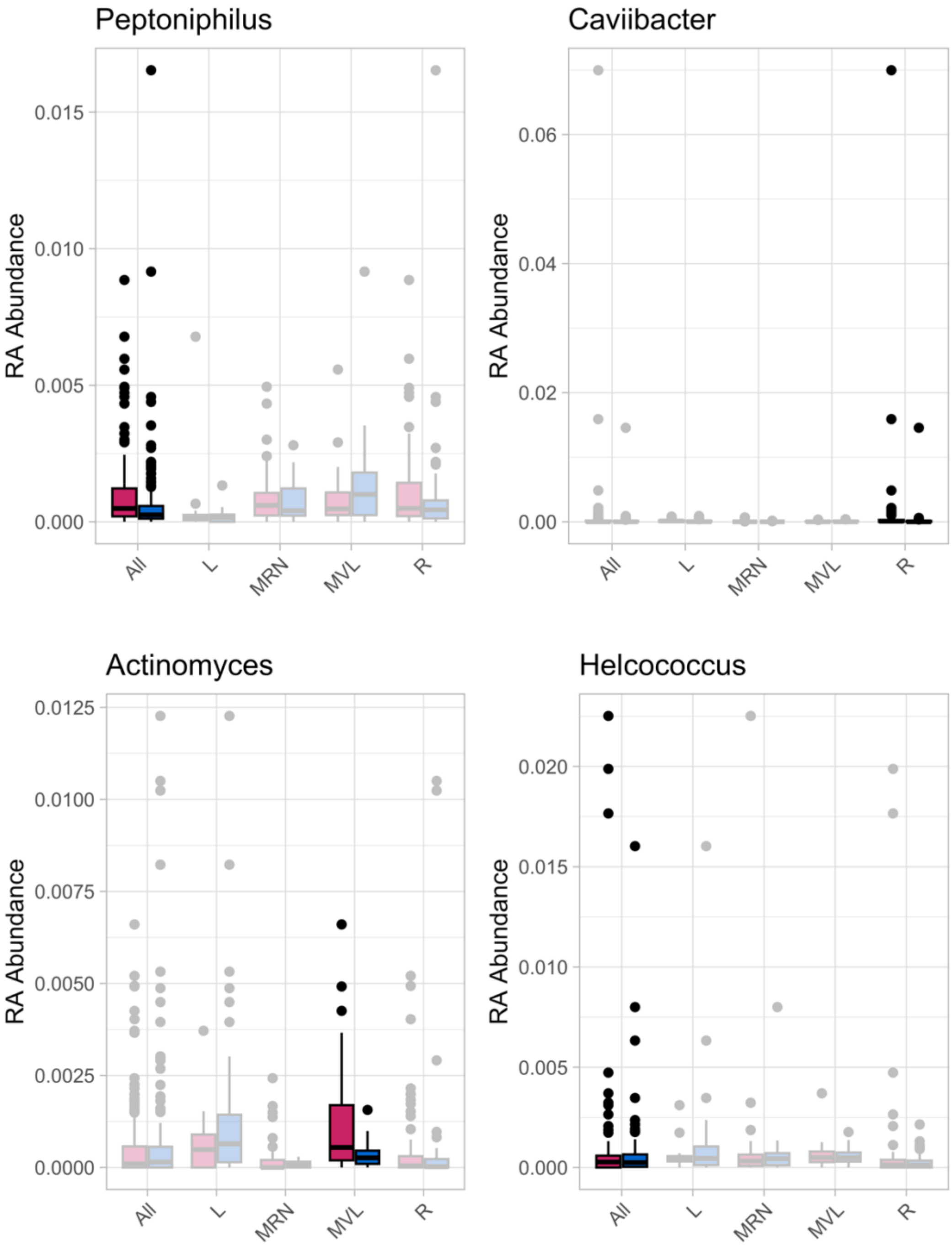

Figure S11. Boxplots of taxa with significant differential abundance between pregnant and non-pregnant ewes for *Peptoniphilus*, *Caviibacter*, *Actinomyces*, and *Helococcus* genera. Pregnancy-positive samples in blue and pregnancy-negative samples in red. The x-axis categorizes samples by herd level. Saturated colors represent groups with statistically significant differences; desaturated colors are used for groups without significant differences.

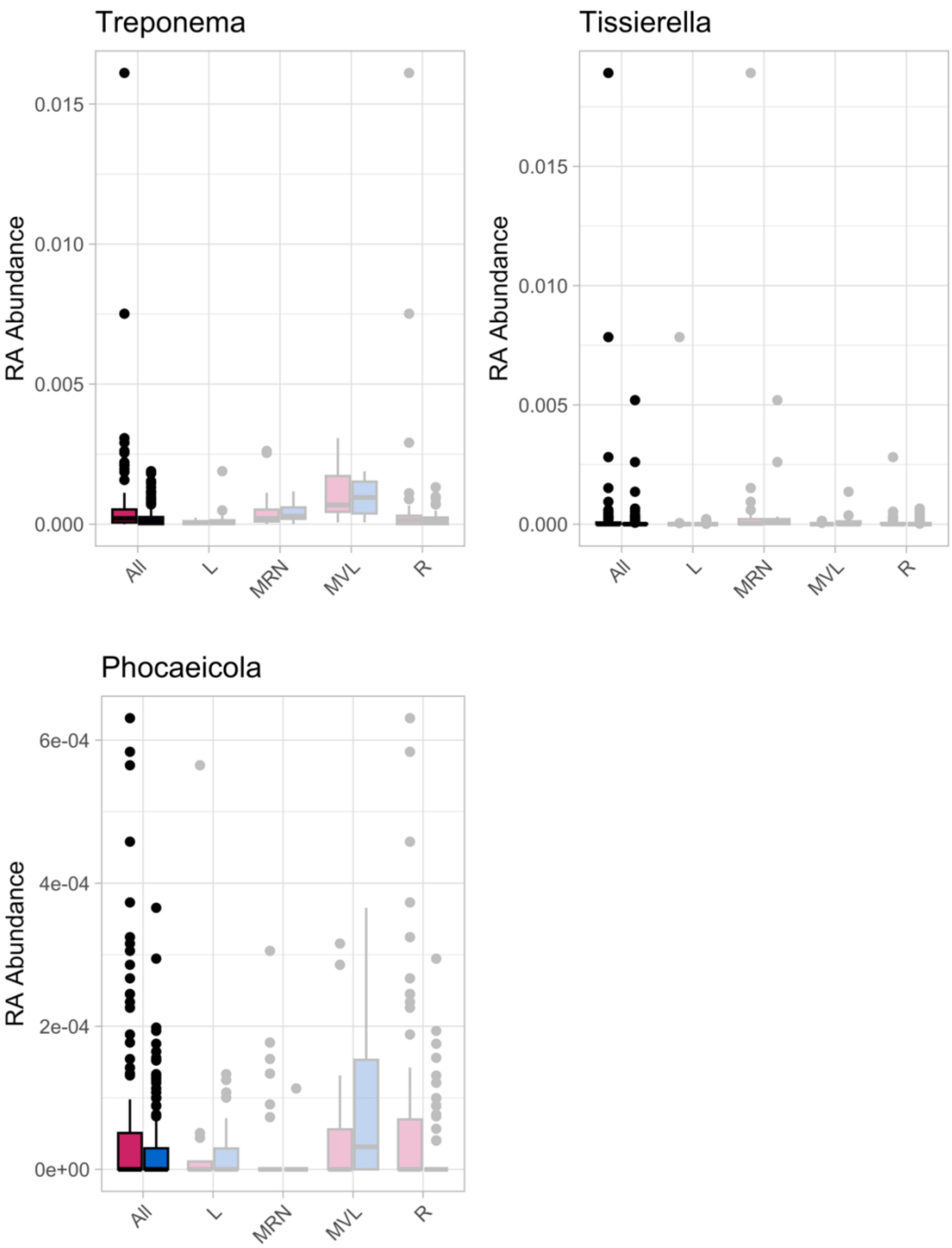

Figure S12. Boxplots of taxa with significant differential abundance between pregnant and non-pregnant ewes for *Treponema*, *Tissierella*, and *Phocaeicola* genera. Pregnancy-positive samples in blue and pregnancy-negative samples in red. The x-axis categorizes samples by herd level. Saturated colors represent groups with statistically significant differences; desaturated colors are used for groups without significant differences.

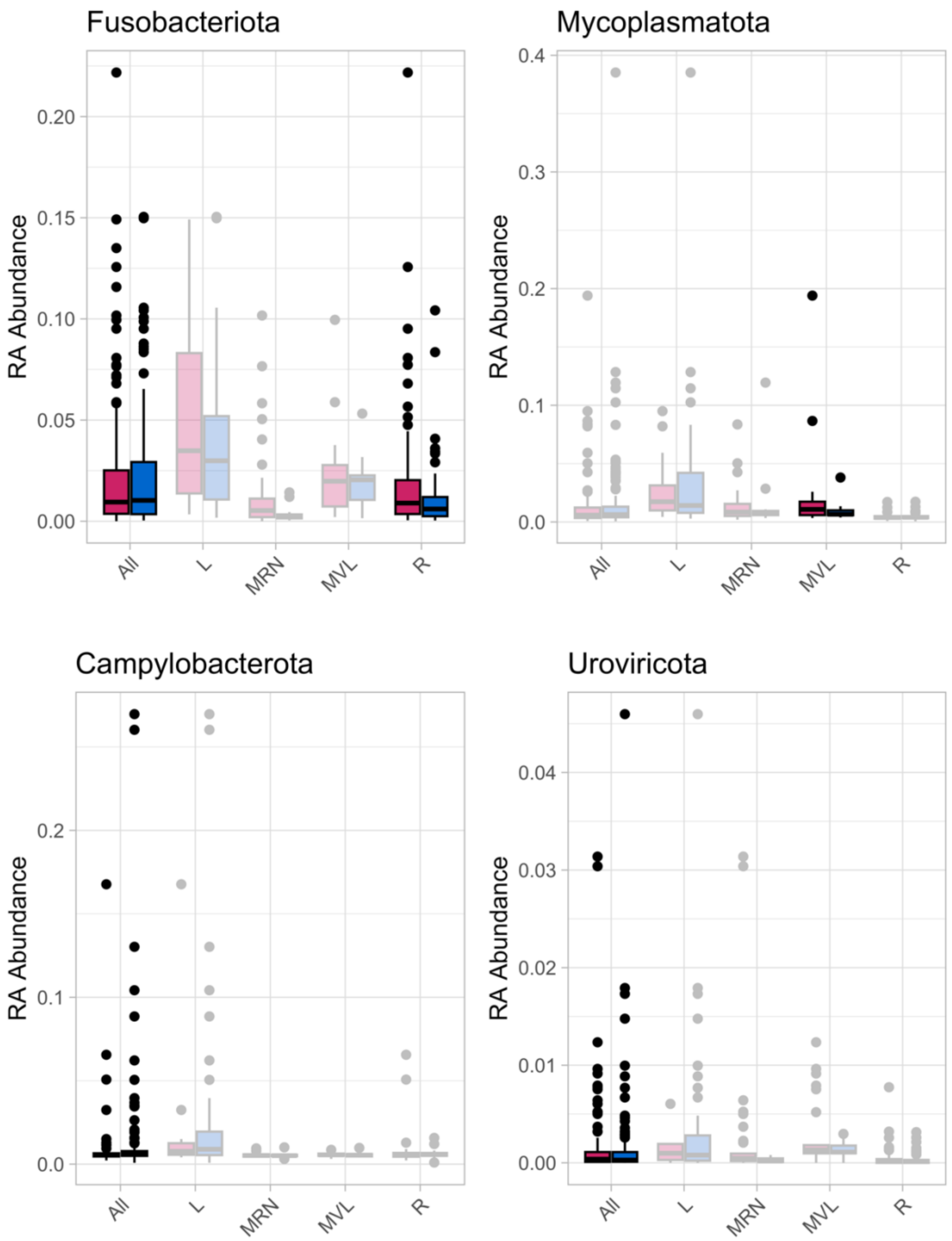

Figure S13. Boxplots of taxa with significant differential abundance between pregnant and non-pregnant ewes for Fusobacteriota, Mycoplasmatota, Campylobacterota, and Uroviricota phyla. Pregnancy-positive samples in blue and pregnancy-negative samples in red. The x-axis categorizes samples by herd level. Saturated colors represent groups with statistically significant differences; desaturated colors are used for groups without significant differences.

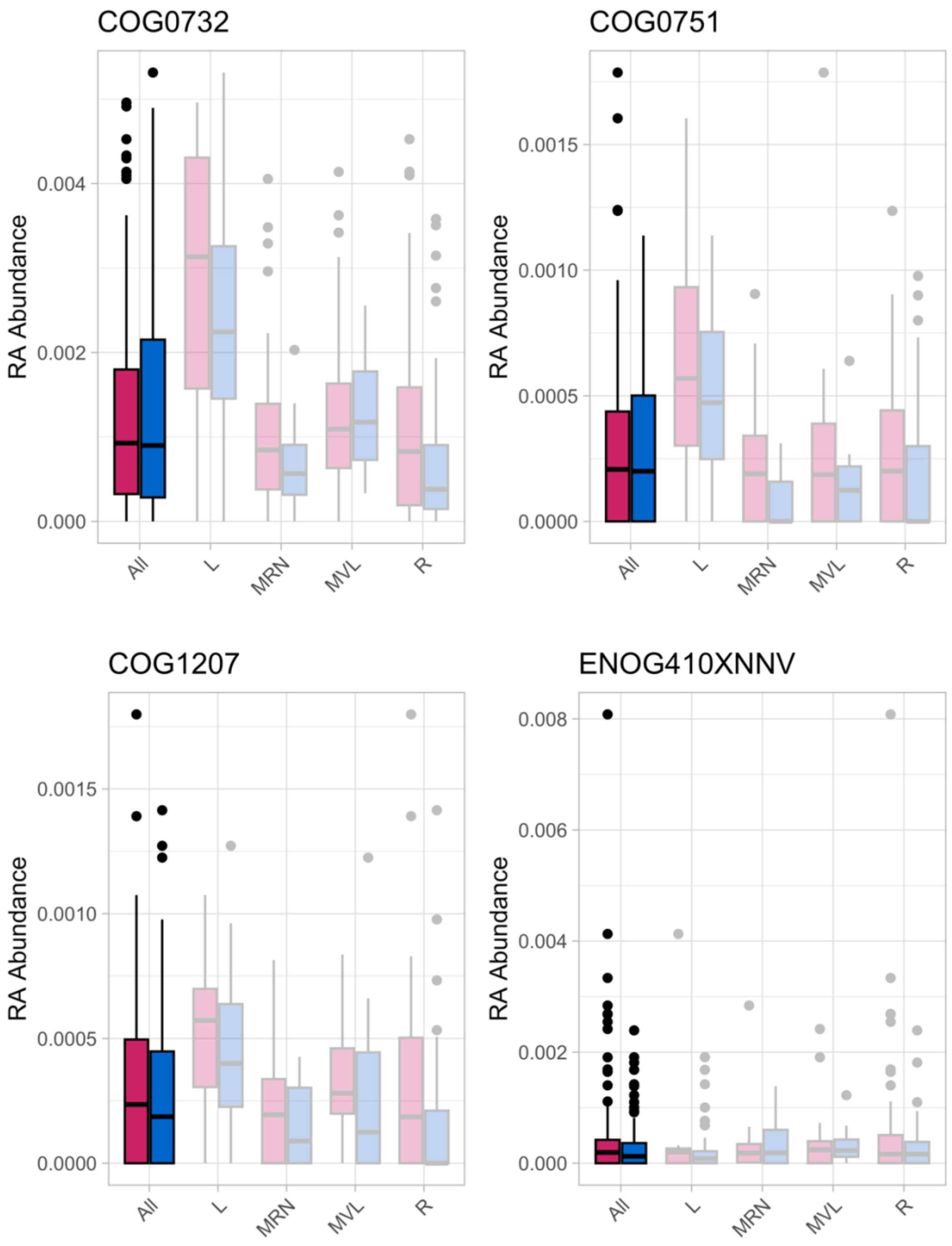

Figure S14. Boxplots of taxa with significant differential abundance between pregnant and non-pregnant ewes for COG0732, COG0751, COG1207, and ENOG410XNNV. Pregnancy-positive samples in blue and pregnancy-negative samples in red. The x-axis categorizes samples by herd level. Saturated colors represent groups with statistically significant differences; desaturated colors are used for groups without significant differences.

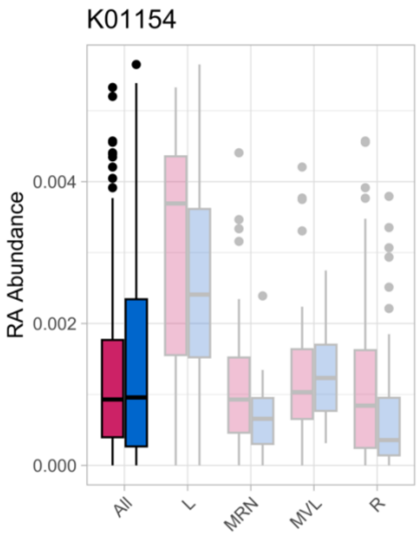

Figure S15. Boxplots of taxa with significant differential abundance between pregnant and non-pregnant ewes for KO1154. Pregnancy-positive samples in blue and pregnancy-negative samples in red. The x-axis categorizes samples by herd level. Saturated colors represent groups with statistically significant differences; desaturated colors are used for groups without significant differences.

Table S1. PERMANOVA results for the effects of Pregnancy, age category, and parity category on microbiota composition within each herd group at genus level.

Variable		SumOfSqs	R2	F	Pr(>F)
Pregnancy		4,394.1	0.010	3.057	**0.001**
	L	643.90	0.010	0.647	0.997
	R	1,164.61	0.007	0.896	0.736
	MRN	1,260.31	0.020	1.074	0.247
	MVL	1,536.83	0.026	1.043	0.285
Age category		1,8897.07	0.031	2.352	**0.001**
	L	4,665.00	0.071	1.160	0.084
	MRN	2,381.40	0.080	1.109	0.170
	MVL	1,863.00	0.099	0.985	0.510
	R	10,027.00	0.038	1.282	**0.035**
Parity category		16,006.77	0.026	1.983	**0.001**
	L	4,779.00	0.072	1.191	0.062
	MRN	1,781.20	0.060	1.104	0.205
	MVL	1,800.50	0.095	0.949	0.666
	R	9,622.00	0.037	1.228	**0.011**

999 permutations were implemented. **SumOfSqs**: Sum of squares, a measure of variance. **R2**: Coefficient of determination, indicating the proportion of variance explained by the factor. **F**: F-statistic, a ratio used to determine the significance of the factor. **Pr(>F)**: P-value, indicating the statistical significance of the factor. Values in bold indicate significant results (P < 0.05).

Table S2. Pairwise comparisons for Herd groups using permutation MANOVAs on a distance matrix

	L	MRN	MVL
MRN	**0.001**	-	-
MVL	**0.001**	**0.001**	-
R	**0.001**	**0.001**	**0.001**

Pairwise comparisons were performed based on euclidean distance matrix at the genus level. 999 permutations were implemented, and p-values were adjusted using the FDR method. Values in bold indicate significant results (adjusted P-value < 0.05).

Table S3. Pairwise comparisons for Age category groups using permutation MANOVAs on a distance matrix

	**< 2 years**	**2-3 years**	**3-4 years**	**4-5 years**
**2-3 years**	0.2810	-	-	-
**3-4 years**	**0.0357**	0.2456	-	-
**4-5 years**	**0.0250**	0.0900	**0.0250**	-
**> 5 years**	**0.0025**	**0.0025**	**0.0025**	**0.0025**

Pairwise comparisons were performed based on euclidean distance matrix at the genus level. 999 permutations were implemented, and p-values were adjusted using the FDR method. Values in bold indicate significant results (adjusted P-value < 0.05).

Table S4. Pairwise comparisons for Parity category groups using permutation MANOVAs on a distance matrix

	< 2	2-3	3-4	4-5
**2-3**	**0.0060**	-	-	-
**3-4**	0.1363	**0.0117**	-	-
**4-5**	**0.0025**	**0.0025**	0.2980	-
**> 5**	**0.0025**	**0.0025**	0.2980	0.06710

Categories represent the number of parturitions. Pairwise comparisons were performed based on euclidean distance matrix at the genus level. 999 permutations were implemented, and p-values were adjusted using the FDR method. Values in bold indicate significant results (adjusted P-value < 0.05).
